# Cationic Axial Ligand Effects on Sulfur-Substituted Subphthalocyanines

**DOI:** 10.3390/molecules27092766

**Published:** 2022-04-26

**Authors:** Yusaku Ogura, Masahiro Nakano, Hajime Maeda, Masahito Segi, Taniyuki Furuyama

**Affiliations:** 1 Graduate School of Natural Science and Technology, Kanazawa University, Kakuma-machi, Kanazawa 920-1192, Japan; rkddm_5658@stu.kanazawa-u.ac.jp (Y.O.); masahiro-nakano@se.kanazawa-u.ac.jp (M.N.); maeda-h@se.kanazawa-u.ac.jp (H.M.); segi@staff.kanazawa-u.ac.jp (M.S.); 2 Japan Science and Technology Agency (JST)-PRESTO, 4-1-8 Honcho, Kawaguchi, Saitama 332-0012, Japan

**Keywords:** subphthalocyanine, axial ligand, absorption spectra, fluorescence, ionization potential

## Abstract

Herein, we report the synthesis of sulfur-substituted boron(III) subphthalocyanines (SubPcs) with cationic axial ligands. Subphthalocyanines were synthesized by a condensation reaction using the corresponding phthalonitriles and boron trichloride as a template. An aminoalkyl group was introduced on the central boron atom; this process was followed by *N*-methylation to introduce a cationic axial ligand. The peripheral sulfur groups shifted the Q band of SubPcs to a longer wavelength. The cationic axial ligands increased the polarity and enhanced the hydrophilicity of SubPcs. The effect of axial ligands on absorption and fluorescence properties is generally small. However, a further red shift was observed by introducing cationic axial ligands into the sulfur-substituted SubPcs. This change is similar to that in sulfur-substituted silicon(IV) phthalocyanines. The unique effect of the cationic axial ligand was extensively investigated by theoretical calculations and electrochemistry. In particular, the precise oxidation potential was determined using ionization potential measurements. Thus, the results of the present study provide a novel strategy for developing functional dyes and pigments based on SubPcs.

## 1. Introduction

**Figure 1:**
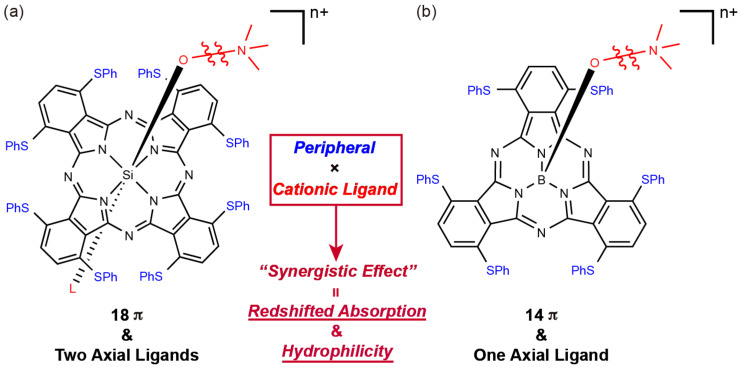
Structures of: (**a**) silicon(IV) phthalocyanine and (**b**) boron(III) subphthalocyanine with cationic axial ligands.

Organic dyes and pigments have intense absorption bands in the visible-to-near-infrared (NIR) regions, demonstrating bright, visible colors. Because the interaction between light and molecules corresponds to electronic transitions, molecules with bright colors often exhibit unique electronic properties [1]. In the last decade, many organic dye-based molecules have been successfully developed and used in the various fields, such as dye-sensitized solar cells [2,3], organic field-effect transistors [4,5], organic photovoltaics (OPVs) [6,7], chemical sensors [8], and photodynamic therapy (PDT) photosensitizers [9,10,11]. Most functional materials require multiple functional features. In novel functional molecules, an individual molecule offers multiple functional sites simultaneously, such as intense light absorption and emission, hydrophilic interaction, and recognition of specific molecules. Molecular platforms that are easily tunable and can integrate various functions are attractive synthetic targets. Among those are phthalocyanines (Pcs), which consist of four isoindole units linked by four nitrogen bridges instead of the carbon in normal porphyrins, and are one of the candidates. Additionally, Pcs have an intense absorption band in the range of 650–700 nm (known as the Q band). Various substituents and elements can be introduced into the peripheral region and the central core. Appropriate modifications of Pcs induce unique structural, optical, electrochemical, magnetic, and biological properties [12,13,14]. These properties can be instigated by introducing various substituents and elements into the peripheral region and the central core of Pcs. Among the reported Pcs, the modification of the two axial ligands of silicon(IV) Pcs (SiPcs) are well established [15]. Although modifying the axial ligands of typical SiPcs marginally affected the Q-band position [16], we recently established an exceptional red shift of the absorption of sulfur-substituted SiPcs by introducing ammonium axial ligands (Figure 1a). The introduction of cationic axial ligands induced a shift of the Q band toward the NIR region and enhanced hydrophilicity. Thus, a combination of peripheral sulfur substituents and cationic axial ligands is applicable to NIR-activatable PDT sensitizers with simple synthetic procedures [17].

In this study, we describe the effect of the cationic axial ligand on sulfur-substituted subphthalocyanines (SubPcs). Subphthalocyanines are Pc congeners containing three isoindole units bridged imino-nitrogen atoms and boron as the central atom. After its first synthesis by Meller and Ossko in 1972 [18], SubPcs generated attention in the field of organic functional dyes, such as in supramolecular chemistry [19,20] and OPVs [21,22]. The unique concave structure and intense fluorescence peaks of SubPcs are advantageous features over that of typical Pcs, suggesting the further potential of SubPcs [23,24,25,26]. In addition, the peripheral substituent effect of SubPcs is similar to that of Pcs; the Q band of sulfur-substituted SubPcs is red-shifted, with the peak appearing at approximately 600 nm [27,28]. Furthermore, SubPc has an easily convertible single axial ligand on the central boron. Hence, we expected the occurrence of the cationic axial ligand effect on sulfur-substituted SubPcs, which is similar to that in SiPcs (Figure 1b).

## 2. Results and Discussion

### 2.1. Synthesis of Subphthalocyanines

**Scheme 1:**
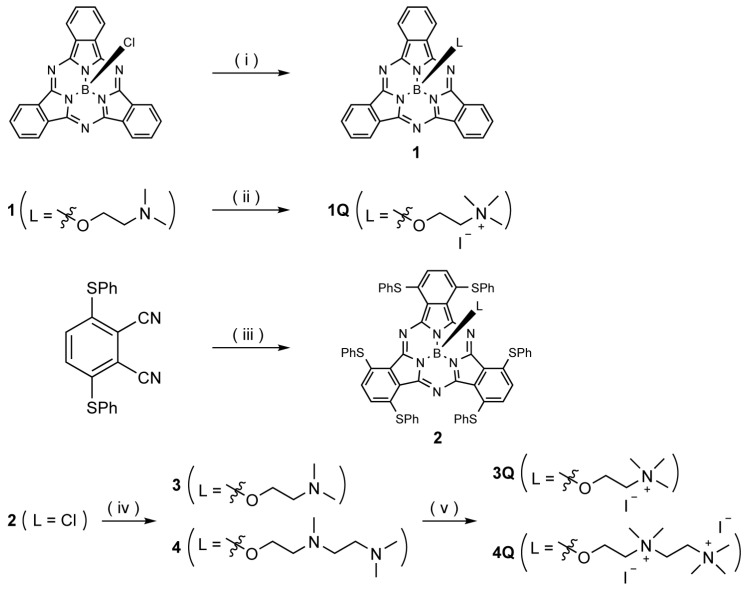
Synthesis of subphthalocyanines (SubPcs). *Reagents and conditions*: (**i**) 2-(dimethylamino)ethan-1-ol, toluene, reflux, 12 h, 19%; (**ii**) iodomethane, CHCl_3_, rt, 1 h, 62% (**iii**) BCl_3_, 1-chloronapthalene, 150 °C, 1 h, 2%; (**iv**) 2-(dimethylamino)ethan-1-ol, 1,2-dichlorobenzene (*o*-DCB), 135 °C, 6 h, 29% (for **3**), 2-[[2-(dimethylamino)ethyl](methyl)amino]ethan-1-ol, *o*-DCB, 135 °C, 8 h, 57% (for **4**); (**v**) iodomethane, CHCl_3_, rt, 1 h, 59% (for **3Q**); iodomethane, CHCl_3_, rt, 7 d, 74% (for **4Q**).

The synthetic route to SubPcs with and without peripheral sulfur-based substituents is shown in Scheme 1. Axial ligands were introduced by a substitution reaction between the chlorine-substituted SubPc and corresponding alcohols. Nitrogen on the axial ligand of **1** was quaternized by *N*-methylation with methyl iodide; the corresponding cation complex **1Q** was obtained in high yield. The sulfur-substituted SubPc **2** was synthesized from 3,6-bis(phenylthio)phthalonitrile using boron trichloride as a template. Subsequently, the aminoalkyl-substituted (**3** and **4**) and ammonium-substituted (**3Q** and **4Q**) macrocycles were prepared in a manner similar to that used to synthesize **1** and **1Q**. The quaternization of the diamino ligand (**4**) was completed in one week, whereas the monoamino ligand (**3**) was quaternized in 1 h. All SubPcs were fully characterized by ^1^H NMR spectroscopy and HR-MALDI-FT-ICR mass spectrometry. Unfortunately, single crystals of SubPcs suitable for X-ray diffraction analysis could not be obtained.

### 2.2. Cationic Axial Ligand Effect of Sulfur-Substituted Subphthalocyanines

**Figure 2:**
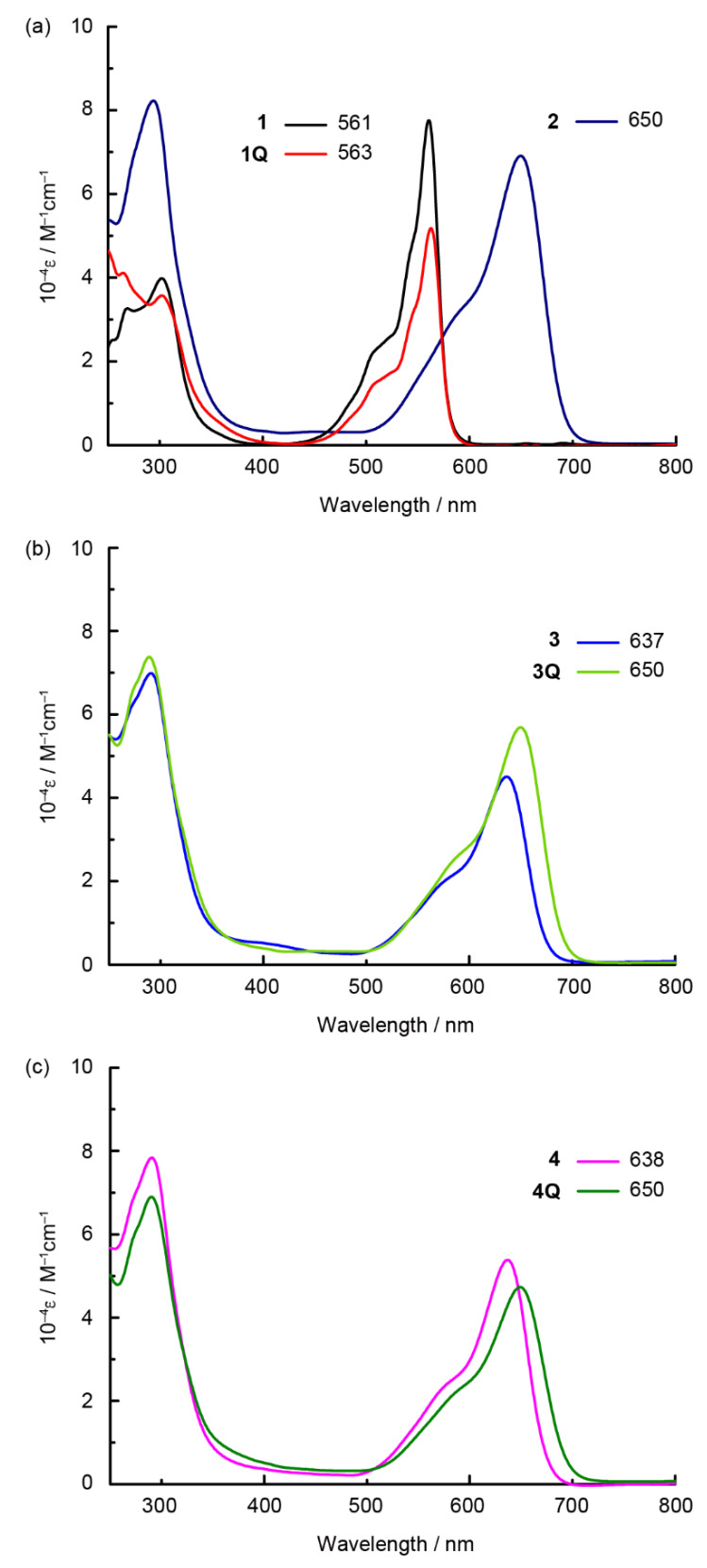
UV-vis-NIR absorption spectra of SubPcs in dichloromethane solutions. (**a**) **1** (black), **1Q** (red), and **2** (purple); (**b**) **3** (blue), and **3Q** (light green); (**c**) **4** (pink) and **4Q** (green).

**Figure 3:**
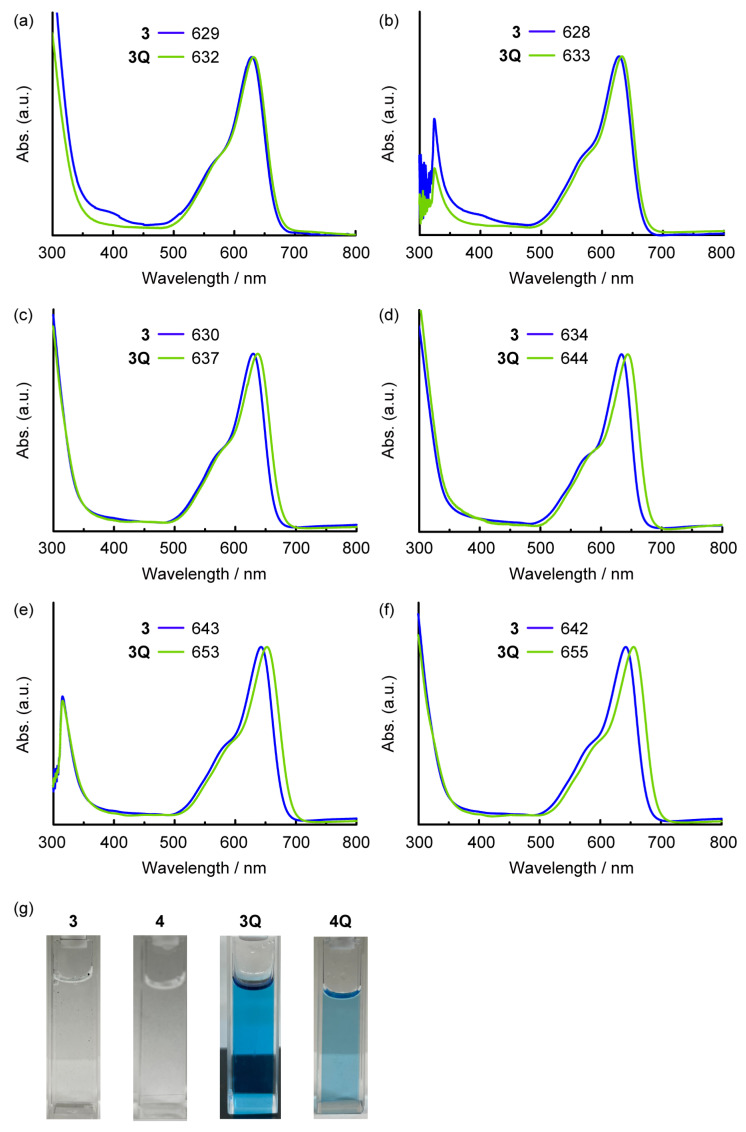
UV-vis-NIR absorption spectra of **3** (blue) and **3Q** (light green) in (**a**) DMSO, (**b**) acetone, (**c**) THF, (**d**) toluene, (**e**) *o*-DCB, and (**f**) CHCl_3_ solutions (*c* = ca. 1.0 × 10^−5^ M). (**g**) Photographs of DMSO/Phosphate-buffered saline (PBS) buffer = 1:1 (*v*/*v*) solutions of **3**, **4**, **3Q**, and **4Q** (*c* = approximately 1.0 × 10^−5^ M). Compounds **3** and **4** were insoluble under this condition.

The absorption spectra of the prepared SubPcs in dichloromethane are shown in Figure 2. The Q band of the unsubstituted SubPc was observed at approximately 550 nm. The intense Q band transition can be assigned to the π–π* transition of the SubPc macrocycle. Because the axial ligand located perpendicular to the SubPc macrocycle did not interact with the π-orbitals of SubPc, it did not affect the position of the Q band. Hence, the Q band positions of **1** (561 nm) and **1Q** (563 nm) were almost identical (2 nm, 60 cm^–1^). By contrast, the Q band was blue-shifted in sulfur-substituted SubPcs when the axial ligand changed from chloride (**2**) to alkyl groups (**3** and **4**). However, the neutral axial ligands did not affect the position of the Q band. Remarkably, a further red shift was observed after *N*-methylation of the axial ligand in **3** (637 nm) to generate **3Q** (650 nm) (13 nm, 310 cm^–1^). This change is similar to the phenomenon observed in sulfur-substituted SiPcs [17]. Although a similar red shift was observed for the dicationic SubPc **4Q**, its Q band position (650 nm) was analogous to that of **3Q**. The effect of the cationic axial ligand on SubPc is independent of the valency of the axial ligand. The effect of solvents on **3** and **3Q** is shown in Figure 3a–f. A significant shift was observed for all solvents (DMSO, acetone, THF, toluene, 1,2-dichlorobenzene (*o*-DCB), and chloroform) that dissolved neutral and cationic SubPcs. Thus, the red shift of **3Q** is not associated with a simple solvent effect. The cationic axial ligands also improved the hydrophilicity of the SubPcs (Figure 3g). SubPcs with neutral aminoalkyl ligands (**3** and **4**) could not be dissolved in model hydrophilic media (DMSO/Phosphate-buffered saline (PBS) buffer = 1:1, *v*/*v*), but the solubility was improved after *N*-methylation of the axial ligands (**3Q** and **4Q**).

**Figure 4:**
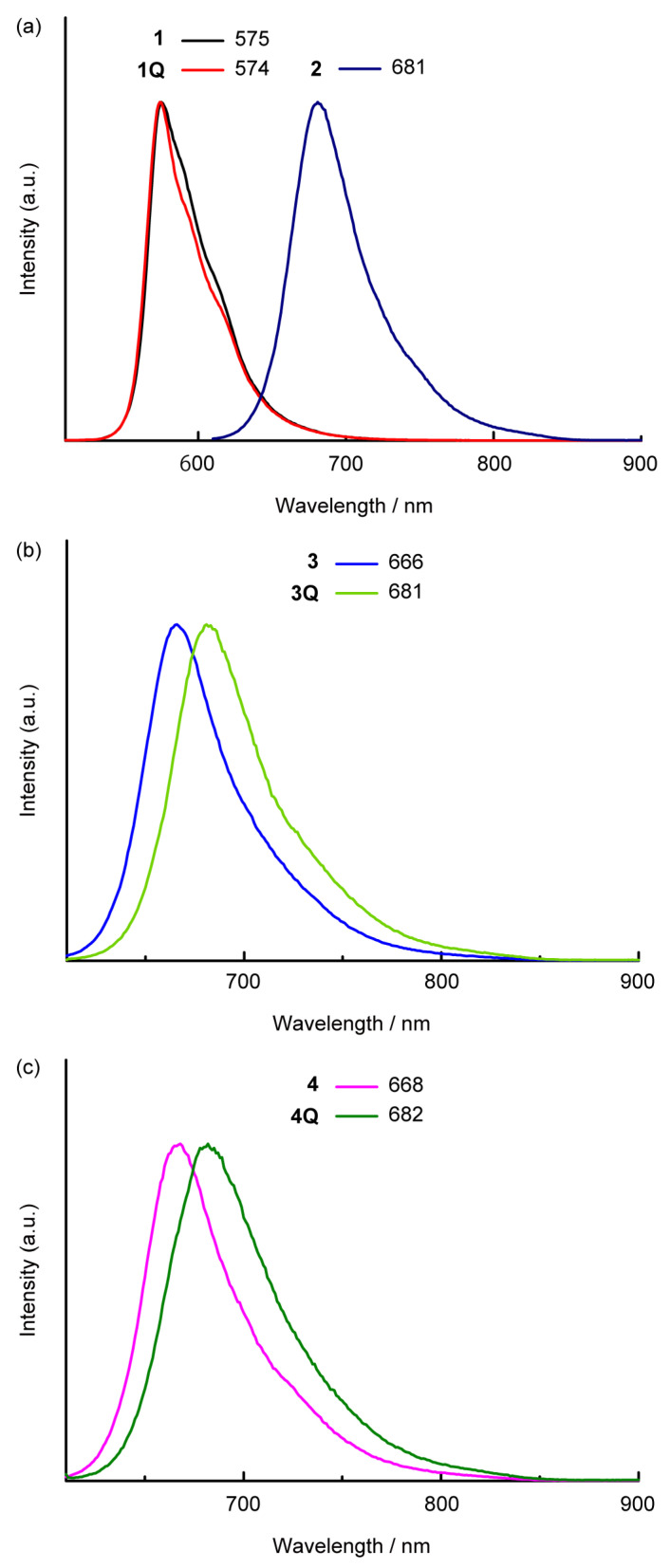
Fluorescence spectra of SubPcs in dichloromethane solutions. (**a**) **1** (black), **1Q** (red), and **2** (purple); (**b**) **3** (blue), and **3Q** (light green); (**c**) **4** (pink) and **4Q** (green). λ_ex_ = 500 nm (for **1** and **1Q**) and 600 nm (for **2**, **3**, **3Q**, **4** and **4Q**).

The fluorescence spectra in dichloromethane are illustrated in Figure 4. Clear fluorescence bands were observed for all SubPcs. The cationization effect of the axial ligand in the sulfur-substituted SubPcs was also observed in the emission properties, whereas a slight difference was observed between the Stokes shift of neutral and cationic compounds. Because the Stokes shift depends on the peripheral substituents, the values of sulfur-substituted SubPcs (approximately 700 cm^–1^) were larger than that of the unsubstituted SubPcs (approximately 400 cm^–1^). Fluorescence quantum yields (Φ_PL_) and lifetimes (*τ*) of SubPcs were measured to obtain further insights into emission properties. The fluorescence parameters are listed in Table 1. The fluorescence quantum yields were almost similar (Φ_PL_s = 0.05–0.07), excluding for **1Q** and **2**. The fluorescence lifetime of **1Q** (*τ* = 1.5 ns) was longer than that of **1** (*τ* = 0.90 ns), suggesting the increase in Φ_PL_ was due to the decrease in the nonradiative decay rate constant (*k*_nr_). The Φ_PL_ of sulfur-substituted SubPc **2** with a chloride axial ligand was high (Φ_PL_ = 0.11), whereas that of other sulfur-substituted SubPcs with alkyl axial ligands was low. Although the fluorescence rate constant (*k*_r_) was almost unchanged, the *k*_nr_ increased, suggesting the decrease in the thermal deactivation of Φ_PL_ due to free rotation of the axial ligand. Remarkably, compared to the unsubstituted SubPcs, sulfur-substituted SubPcs’ emission properties did not change significantly when the axial ligands were changed from neutral to cationic. Although the peripheral substituent effects on the emission properties are estimated to be more significant than the axial ligand effects, the details are still unclear within the scope of these analyses.

### 2.3. Electrochemical Properties

**Figure 5:**
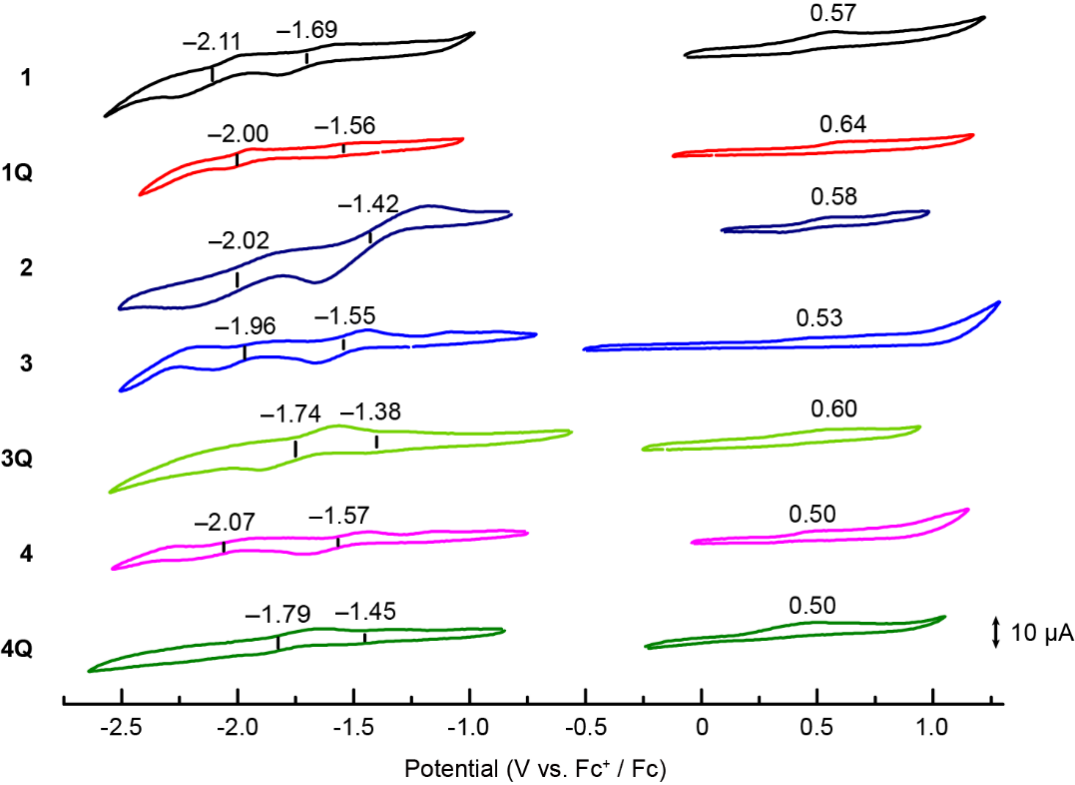
Cyclic voltammograms of **1** (black), **1Q** (red), **2** (purple), **3** (blue), **3Q** (light green), **4** (pink), and **4Q** (green) recorded from 1.0 mM solutions of the analytes in [*^n^*Bu_4_N]ClO_4_/*o*-DCB. Ferrocene was used as the internal standard, and the Fc/Fc^+^ couple was set to 0 V.

**Figure 6:**
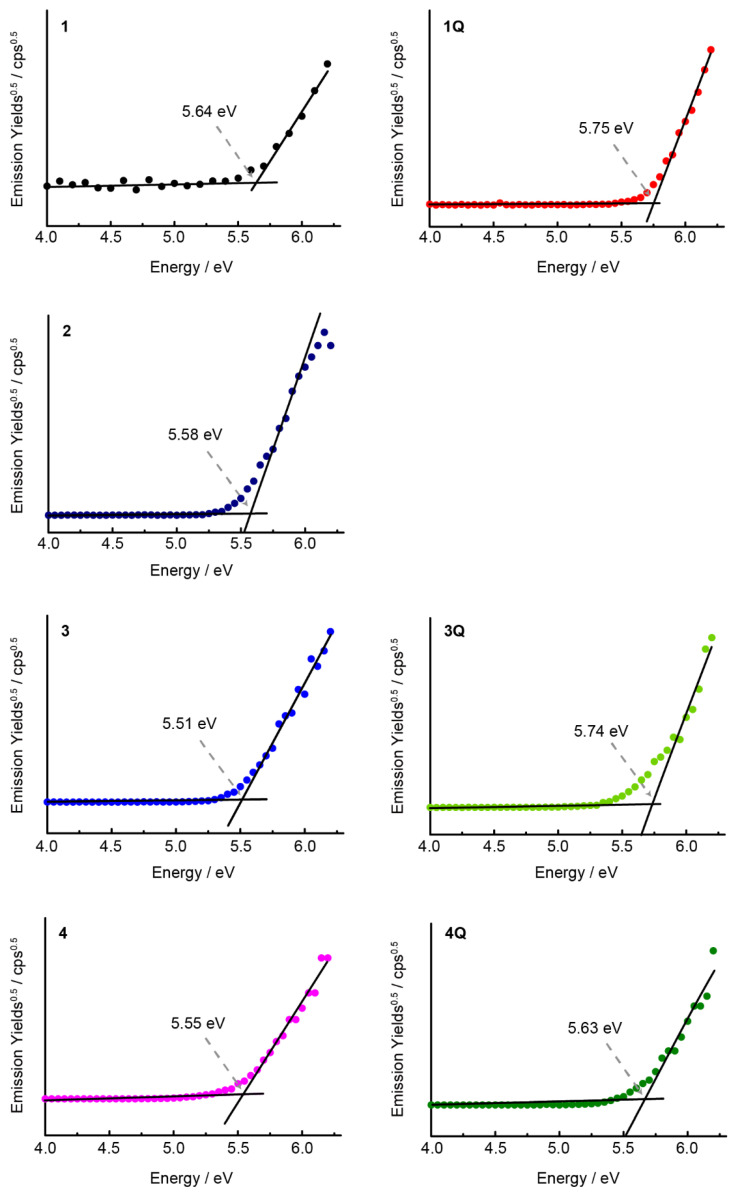
Photoelectron spectra of spin-coated thin films of SubPcs on an ITO glass substrate.

Cyclic voltammograms were recorded in *o*-DCB (Figure 5) to gain insight into the origin of the cationic axial ligand effect of SubPcs. The highest occupied molecular orbital (HOMO) and lowest unoccupied molecular orbital (LUMO) energies of Pc derivatives correlate well with their first oxidation and reduction potentials [29,30]. In the case of unsubstituted SubPcs, the redox potentials shifted anodically when the neutral axial ligand (**1**) was substituted by the cationic axial ligand (**1Q**), indicating the stabilization of the molecular orbitals of SubPc by the electron-deficient cationic moiety. However, the difference between the first oxidation and reduction potentials (*E*_1ox_–*E*_1red_) did not change significantly, supporting the absorption spectrum results. When sulfur groups were introduced into the peripheral position, the reduction potential shifted anodically, similar to that in Pcs [28,31], resulting in a decrease in the *E*_1ox_–*E*_1red_ values. Furthermore, the effect on the cationization of the axial ligand was similar to that of unsubstituted SubPc. The first reduction potential of **3Q** at −1.38 V corresponds to the anodic shift of 0.17 V upon *N*-methylation, whereas the first oxidation potential at 0.60 V corresponds to the anodic shift of 0.07 V. Therefore, the value of *E*_1ox_–*E*_1red_ was decreased further, which was observed for the sulfur-substituted SiPcs [17]. Conversely, the difference between the monocationic SubPc (**3Q**) and dicationic SubPc (**4Q**) was insignificant. Although these results nearly describe the changes in the absorption spectra, the difficulty in determining the precise oxidation potential using cyclic voltammetry (or differential pulse voltammetry) remains persistent, which is a common problem for SubPcs [32]. The oxidation wave was irreversible for all compounds, and no evident wave was obtained, indicating that the changes in oxidation potential could not be compared precisely. Therefore, we measured the ionization potentials of SubPc as a direct method to estimate the HOMO level. Ionization potentials were measured on spin-coated SubPc films on indium tin oxide substrates (Figure 6). The obtained ionization potentials correlate with the HOMO, which is estimated from the oxidation potentials of the cyclic voltammograms [33]. The ionization potential of the unsubstituted **1** (−5.64 eV) was larger than that of the sulfur-substituted SubPcs **2**–**4** (−5.51–−5.58 eV), indicating the destabilization of the HOMOs due to the introduction of electron-donating sulfur groups. The potentials of **1Q** (−5.75 eV), **3Q** (−5.74 eV), and **4Q** (−5.63 eV) with cationic axial ligands increased compared to the corresponding neutral compounds, which also supports the absorption spectra and cyclic voltammograms. However, the change in the potential from **4** to **4Q** (0.08 eV) was smaller than that from **3** to **3Q** (0.23 eV) because the reduction potential of **4Q** (−1.45 V) appeared at the cathode side as compared to **3Q** (−1.38 V). In particular, the stabilization of molecular orbitals in the dication compound is smaller than that in the monocation compound, and the apparent absorption spectra between **3Q** and **4Q** are almost similar.

### 2.4. Molecular Orbital Calculations

**Figure 7:**
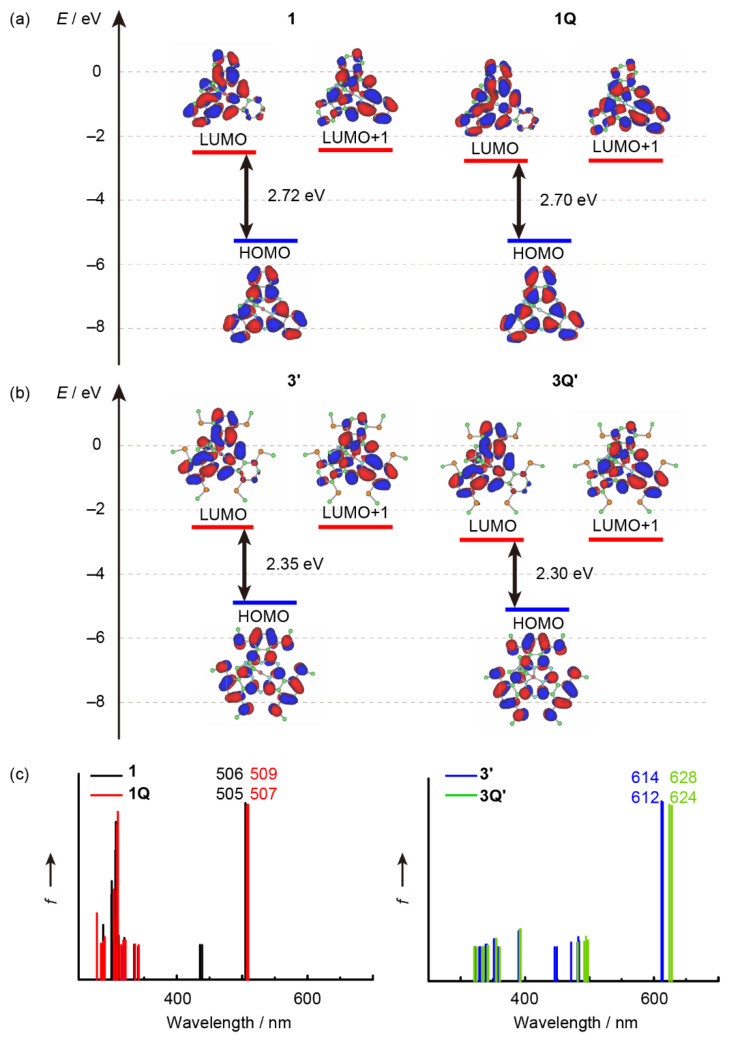
Partial molecular energy diagram and orbitals of (**a**) peripherally unsubstituted SubPcs (**1** and **1Q**), (**b**) peripherally substituted (MeS)_6_SubPcs (**3′** and **3Q′**), and (**c**) their calculated absorption spectra. Calculations were performed at the B3LYP/6-31G* level of theory, using a polarizable continuum model (PCM), which mimicked the solvation effect of dichloromethane. (Details in Supplementary Material).

Based on the experimental optical and electrochemical results, we rationalized the cationic axial ligand effects on SubPcs by performing molecular orbital (MO) calculations. According to previous studies, the effect of sulfur atoms is much higher than that of the substituent groups on sulfur atoms [28,31,34]. Because the effect of the phenyl groups on the absorption spectra was insignificant, model structures **3′** and **3Q′** were used, where the phenyl groups on the sulfur atoms were replaced by the methyl groups. The partial MO energy diagrams of the unsubstituted SubPcs (**1** and **1Q**) and model sulfur-substituted SubPcs (**3′** and **3Q′**) along with the calculated absorption spectra are illustrated in Figure 7. The results of time-dependent density functional theory (TD-DFT) calculations are listed in Table 2. We also conducted TD-DFT calculations using the ωB97XD/6-31G* (Table S1 and Figure S1). The trend of the stick absorption spectra and the energy level of frontier orbitals are identical. However, the calculated absolute values of transition wavelengths were worse than the B3LYP/6-31G*. The B3LYP/6-31G* is suitable for explaining electronic structures in this study. The envelopes of the frontier orbitals of the SubPc ligand were almost identical, and the calculated transitions in the Q-band region were composed of the HOMO, LUMO, and LUMO+1; LUMO and LUMO+1 and almost degenerated. These features are typical characteristics of the 14π aromatic macrocycle, indicating that these absorption bands can be described similarly to that of typical SubPcs even after introducing cationic axial ligands. The energy levels of the frontier orbitals were completely stabilized for the change from **1** to **1Q**. The HOMO–LUMO energy gap (ΔHL) (**1**: 2.72 eV, **1Q**: 2.70 eV) and calculated absorption wavelengths (**1**: 506 nm, **1Q**: 509 nm) are almost identical, indicating that the cationic axial ligand does not affect the envelope of the absorption spectrum in typical unsubstituted SubPcs. A similar stabilization of the frontier orbitals was estimated for the change from **3′** to **3Q′**, whereas the ΔHL decreased from the neutral **3′** (2.35 eV) to cation **3Q′** (2.30 eV). Moreover, the calculated absorption peak of **3Q′** (628 nm) was longer than **3′** (614 nm). When cationic axial ligands were introduced in sulfur-substituted SubPcs, the LUMO stabilization was higher (ΔLUMO: 0.26 eV) than that of HOMO (ΔHOMO: 0.21 eV), which was consistent with the experimental results of the optical and electrochemical measurements. A small MO contribution at the central boron was observed for the frontier orbitals of all the calculated SubPcs, indicating that the cationic axial ligand effect could be assigned to the inductive effect of an electron-deficient cationic moiety, as previously observed for phosphorus(V) phthalocyanines [13,31]. Additionally, the peripheral sulfur substituents can synergistically enhance the axial ligand effect.

## 3. Materials and Methods

### Synthesis of Subphthalocyanines

3,6-Bis(phenylthio)phthalonitrile was synthesized according to published procedures [31]. Boron subphthalocyanine chloride was purchased from Merck KGaA.

Unsubstituted SubPc (Axial: 2-(dimethylamino)ethoxy) (**1**) [35]: A mixture of boron subphthalocyanine chloride (purity: ca. 85%, 115 mg, 0.23 mmol) and 2-(dimethylamino)ethan-1-ol (0.57 mL, 5.7 mmol) in toluene (6.0 mL) was heated for 12 h under reflux. After cooling, the volatiles were removed under reduced pressure. The residue was then loaded onto a silica gel column and eluted with CHCl_3_/MeOH (from 40:1 (*v*/*v*) to 20:1 (*v*/*v*)). After evaporation of the solvent *in vacuo*, the precipitate was recrystallized from acetone/hexane to give **1** as a violet powder (21 mg, 19%).

400 MHz ^1^H NMR(CDCl_3_) δ (ppm): 8.87–8.82 (m, 6H, SubPc-H), 7.91–7.86 (m, 6H, SubPc-H), 1.69 (s, 6H, CH_3_), 1.54 (t, *J* = 6.2 Hz, 2H, CH_2_), 1.40 (t, *J* = 6.2 Hz, 2H, CH_2_). UV-vis (CH_2_Cl_2_) λ_max_ nm (ε × 10^−4^): 561 (7.8), 302 (4.0), 267 (3.2). λ_PL, max_ (CH_2_Cl_2_): 575 nm, Φ_PL_ = 0.070.

Unsubstituted SubPc (Axial: 2-(trimethylammonium)ethoxy) (**1Q**) [35]: A mixture of **1** (10 mg, 0.020 mmol) and iodomethane (1.0 mL) in chloroform (10 mL) was stirred at room temperature for 1 h. After evaporation of the solvent *in vacuo*, the precipitate was washed with diethyl ether, and then dried in vacuo to give **1Q** as a violet powder (8.0 mg, 62%).

500 MHz ^1^H NMR(CDCl_3_) δ (ppm): 8.89–8.85 (m, 6H, SubPc-H), 7.97–7.93 (m, 6H, SubPc-H), 2.86–2.84 (m, 2H, CH_2_), 2.71 (s, 9H, CH_3_), 1.88 (brs, 2H, CH_2_). UV-vis (CH_2_Cl_2_) λ_max_ nm (ε × 10^−4^): 563 (5.2), 302 (3.6), 265 (4.2). λ_PL, max_ (CH_2_Cl_2_): 574 nm, Φ_PL_ = 0.11.

αα-Hexa-(phenylthio)-SubPc (Axial: chloride) (**2**) [28]: BCl_3_ in *p*-xylene (1.0 mL, 1.0 mmol) was placed in a mixture of a solution of 3,6-bis(phenylthio)phthalonitrile (344 mg, 1.0 mmol) and dry 1-chloronaphthalene (3.0 mL) under argon. The reaction mixture was heated to 150 °C for 1 h. The reaction mixture was poured onto a vigorously stirred cold mixture of MeOH and hexane (20 mL, *v*/*v*, 1:1). The precipitate was submitted to column chromatography on silica gel using chloroform as eluent and the resulting blue solid was washed with methanol, followed by GPC-HPLC (CHCl_3_) to give **2** as a blue powder (8.7 mg, 2%).

500 MHz ^1^H NMR(CDCl_3_) δ (ppm): 7.78–7.74 (m, 12H, Ar-H), 7.46–7.41 (m, 18H, Ar-H), 7.01 (s, 6H, SubPc-H). UV-vis (CH_2_Cl_2_) λ_max_ nm (ε × 10^−4^): 650 (6.9), 294 (8.2). λ_PL, max_ (CH_2_Cl_2_): 681 nm, Φ_PL_ = 0.11.

αα-Hexa-(phenylthio)-SubPc (Axial: 2-(dimethylamino)ethoxy) (**3**): A mixture of **2** (25 mg, 0.023 mmol) and 2-(dimethylamino)ethan-1-ol (58 μL, 0.58 mmol) in 1,2-dichlorobenzene (2.0 mL) was heated to 135 °C for 6 h. After cooling down to room temperature, the solvent was submitted to column chromatography on a silica gel column and eluted with CHCl_3_/MeOH [changing gradually from 100:0 (*v*/*v*) to 20:1 (*v*/*v*)]. The resulting blue solid was washed with methanol to give **3** as a blue powder (7.7 mg, 29%).

400 MHz ^1^H NMR(CDCl_3_) δ (ppm): 7.76–7.72 (m, 12H, Ar-H), 7.45–7.38 (m, 18H, Ar-H), 7.00 (s, 6H, SubPc-H), 1.76 (s, 6H, CH_3_), 1.63–1.62 (m, 2H, CH_2_), 1.49 (brs, 2H, CH_2_). UV-vis (CH_2_Cl_2_) λ_max_ nm (ε × 10^−4^): 637 (4.5), 291 (7.0). λ_PL, max_ (CH_2_Cl_2_): 666 nm, Φ_PL_ = 0.064. HR-MALDI-FT-ICR-MS calcd for C_60_H_36_BN_6_S_6_ [M – L]^+^: 1043.1423. Found: 1043.1409.

αα-Hexa-(phenylthio)-SubPc (Axial: 2-(trimethylammonium)ethoxy) (**3Q**): Synthesized according to the procedure for **1Q**. Blue powder. (4.0 mg, 59%)

500 MHz ^1^H NMR(CDCl_3_) δ (ppm): 7.77–7.74 (m, 12H, Ar-H), 7.47–7.43 (m, 18H, Ar-H), 7.03 (s, 6H, SubPc-H), 2.91–2.89 (m, 2H, CH_2_), 2.80 (s, 9H, CH_3_), 2.00 (brs, 2H, CH_2_). UV-vis (CH_2_Cl_2_) λ_max_ nm (ε × 10^−4^): 650 (5.7), 289 (7.4). λ_PL, max_ (CH_2_Cl_2_): 681 nm, Φ_PL_ = 0.056. HR-MALDI-FT-ICR-MS calcd for C_65_H_49_BN_7_OS_6_ [M – I]^+^: 1146.2421. Found: 1146.2386.

αα-Hexa-(phenylthio)-SubPc (Axial: 2-[[2-(dimethylamino)ethyl]methylamino]ethoxy) (**4**): Synthesized according to the procedure for **3**. Blue powder. (9.5 mg, 57%)

500 MHz ^1^H NMR (CDCl_3_) δ (ppm): 7.76–7.73 (m, 12H, Ar-H), 7.45–7.40 (m, 18H, Ar-H), 6.98 (s, 6H, SubPc-H), 2.10 (s, 6H, CH_3_), 2.08–2.03 (m, 4H, CH_2_), 1.77 (s, 3H, CH_3_) 1.65–1.56 (m, 4H, CH_2_). UV-vis (CH_2_Cl_2_) λ_max_ nm (ε × 10^−4^): 638 (5.4), 290 (7.8). λ_PL, max_ (CH_2_Cl_2_): 668 nm, Φ_PL_ = 0.073. HR-MALDI-FT-ICR-MS calcd for C_60_H_36_BN_6_S_6_ [M – L]^+^: 1043.1423. Found: 1043.1415.

αα-Hexa-(phenylthio)-SubPc (Axial: 2-[[2-(trimethylammonium)ethyl]dimethylammonium]ethoxy) (**4Q**): Synthesized according to the procedure for **1Q**. Blue powder. (5.5 mg, 74%)

400 MHz ^1^H NMR(CDCl_3_) δ (ppm): 7.77–7.73 (m, 12H, Ar-H), 7.47–7.43 (m, 18H, Ar-H), 7.05 (s, 6H, SubPc-H), 4.32–4.28 (m, 2H, CH_2_) 4.02 (brs, 2H, CH_2_), 3.53 (s, 9H, CH_3_), 3.01 (s, 6H, CH_3_), 2.91 (brs, 2H, CH_2_), 2.04 (brs, 2H, CH_2_). UV-vis (CH_2_Cl_2_) λ_max_ nm (ε × 10^−4^): 650 (4.7), 291 (6.9). λ_PL, max_ (CH_2_Cl_2_): 682 nm, Φ_PL_ = 0.053. HR-MALDI-FT-ICR-MS calcd for C_68_H_56_BN_8_OS_6_ [M – 2I – CH_3_]^+^: 1203.3000. Found: 1203.2981.

## 4. Conclusions

In summary, sulfur-substituted SubPcs with cationic axial ligands were synthesized. The macrocyclization of SubPc from the sulfur-substituted phthalonitrile, introduction of axial ligands, and quaternization of axial ligands could be performed according to the method used for unsubstituted SubPcs and SiPcs. The hydrophilicity of cationic SubPcs **3Q** and **4Q** was enhanced, suggesting their potential applications to amphiphilic materials and biology. The absorption spectra of unsubstituted SubPcs **1** and **1Q** were almost unchanged by varying the axial ligand. By contrast, the sulfur-substituted SubPcs demonstrated a further red shift of the Q band by the cationization of the axial ligands. The shift in the emission wavelength and absorption spectra were also observed. No significant change in the Φ_PL_ and *τ* indicates that the fluorescence wavelength can be varied by cationization of the axial ligand without changing the emission properties. The electrochemical measurements showed that the cationization of the axial ligand completely stabilized the frontier MOs. The cationization remarkably changed the reduction potential of the sulfur-substituted SubPcs. Conversely, owing to the instability of the oxidized species of SubPcs, we were unable to observe an evident oxidation wave. Nevertheless, this problem was overcome by ionization potential measurements. The observed ionization potentials described the absorption and cyclic voltammogram measurements well. The calculated transitions using MO calculations reproduced the experimental results, which also indicated that introducing peripheral and cationic axial ligands did not change the aromaticity of SubPc. The MO diagrams indicated the selective stabilization of the LUMO level due to the introduction of peripheral sulfur moieties. These results conclude that a synergistic effect [31] of peripheral and axial substituents can occur in SubPcs. This strategy shows that a single functional group can add multiple functions in SubPcs. Thus, integrating various functions into the unique structural and emission properties of SubPcs allows the development of novel functional materials.

## Figures and Tables

**Table 1:** Optical parameters for SubPcs dichloromethane solutions.

Compound	λ_abs_/nm	λ_ex_/nm	λ_fl_/nm	Φ_PL_	*τ*/ns	*k*_r_/×10^7^ s^−1^	*k*_nr_/×10^8^ s^−1^
**1**	561	500	575	0.07	0.90	7.7	10
**1Q**	563	500	574	0.11	1.5	7.5	5.9
**2**	650	600	681	0.11	1.4	7.8	6.3
**3**	637	600	666	0.06	1.0	6.4	9.4
**3Q**	650	600	681	0.06	0.99	5.6	9.5
**4**	638	600	668	0.07	0.92	7.9	10
**4Q**	650	600	682	0.05	0.80	6.6	12

**Table 2:** Calculated excited wavelengths (λ) and oscillator strengths (*f*) for components of selected transition energies. Calculations were performed at the B3LYP/6-31G* level of theory using a polarizable continuum model (PCM), which mimicked the solvation effect of dichloromethane.

	λ/nm	f	Composition
**1**	506	0.41	HOMO–4→LUMO+1 (2.4%), HOMO→LUMO (96.8%)
	505	0.41	HOMO–4→LUMO (2.5%), HOMO→LUMO+1 (96.7%)
**1Q**	509	0.41	HOMO–3→LUMO+1 (2.5%), HOMO→LUMO (96.8%)
	507	0.41	HOMO–3→LUMO (2.5%), HOMO→LUMO+1 (96.7%)
**3′**	614	0.43	HOMO→LUMO (98.2%)
	612	0.43	HOMO→LUMO+1 (98.2%)
**3Q′**	628	0.42	HOMO→LUMO (98.4%)
	624	0.42	HOMO→LUMO+1 (98.4%)

## Data Availability

The data presented in this study are available in Supplementary Material.

## Supplementary Materials

The following supporting information can be downloaded at: https://www.mdpi.com/article/10.3390/molecules27092766/s1. Table S1: Calculated excited wavelengths (λ) and oscillator strengths (f) for components of selected transition energies. Calculations were performed at the ωB97XD/6-31G*//B3LYP/6-31G* level of theory using a polarizable continuum model (PCM), which mimicked the solvation effect of dichloromethane; Figure S1: Partial molecular energy diagram and orbitals of (a) peripherally unsubstituted SubPcs (**1** and **1Q**), (b) peripherally substituted (MeS)_6_SubPcs (**3****’** and **3Q****’**), and (c) their calculated absorption spectra. Calculations were performed at the ωB97XD/6-31G*//B3LYP/6-31G* level of theory, using a polarizable continuum model (PCM), which mimicked the solvation effect of dichloromethane [36,37,38,39,40,41,42,43,44].

## Sample Availability

Samples of the SubPc compounds are available from the authors.
