# Supplementary material for: Cationic Axial Ligand Effects on Sulfur-Substituted Subphthalocyanines
Source: Molecules. 2022 Apr 26;27(9):2766. doi: 10.3390/molecules27092766 (PMC9105831; doi:10.3390/molecules27092766)
Supplement: Supplementary file 1 [file molecules-27-02766-s001.zip › molecules-1669216-supplementary.pdf]

Cationic Axial Ligand Effects on Sulfur-Substituted Subphthalocyanines

Yusaku Ogura [1], Masahiro Nakano [1], Hajime Maeda [1], Masahito Segi [1] and Taniyuki Furuyama [1,2,*]

[1] Graduate School of Natural Science and Technology, Kanazawa University, Kakuma-machi, Kanazawa 920-1192, Japan; rkddm_5658@stu.kanazawa-u.ac.jp (Y.O.); masahiro-nakano@se.kanazawa-u.ac.jp (M.N.); maeda-h@se.kanazawa-u.ac.jp (H.M.); segi@staff.kanazawa-u.ac.jp (M.S.)

[2] Japan Science and Technology Agency (JST)-PRESTO, 4-1-8 Honcho, Kawaguchi, Saitama 332-0012, Japan

* Correspondence: tfuruyama@se.kanazawa-u.ac.jp

Supplementary Materials

Table of Contents

General Comments S–1

Copies of the NMR Spectra of Studied Compounds S–2

Full Computational Details S–6

General Comments

Instrumentation

NMR spectra were obtained using JEOL ECA-500 or Bruker AVANCE 400 spectrometer. Chemical shifts are expressed in δ (ppm) values, and coupling constants are expressed in hertz (Hz). ^{1}H NMR spectra were referenced to the tetramethylsilane (TMS) as an internal standard. The following abbreviations are used: s = singlet, d = doublet, m = multiplet, and brs = broad singlet. High-resolution mass spectra (HRMS) were recorded using a Bruker Daltonics solariX spectrometer (MALDI). Electronic absorption spectra were recorded on a JASCO V-770 spectrophotometer. Fluorescence spectra were obtained using a HITACHI F-4500 spectrofluorometer. Absolute fluorescence quantum yields (Φ_{PL}) were measured using a calibrated integrating sphere system (Quantaurus-QY Plus C13534-22, Hamamatsu photonics). Time-resolved photoluminescence lifetime (τ) measurements were carried out using a time-correlated single photon counting lifetime spectroscopy system (Quantaurus-Tau C11367-22, Hamamatsu photonics). The decay constants and fitting parameters for the transient decays were determined using the software embedded in the Quantaurus-Tau system. Cyclic voltammetry (CV) measurements were recorded using a Hokuto Denko HZ7000 potentiostat under a nitrogen atmosphere with 0.1 M of tetrabutylammonium perchlorate (TBAP) as the supporting electrolyte. Measurements were made using a glassy carbon electrode (area = 0.07 cm^2), an Ag/AgCl reference electrode, and a Pt wire counter electrode. The concentration of the solution was fixed at 0.5 mM, and the sweep rate was set to 100 mV/s. The ferrocenium/ferrocene (Fc^+/Fc) couple was used as an internal standard. Ionization potentials were determined from the onset of the photoelectron spectroscopy in air (AC-2, Riken Keiki).

Copies of the NMR Spectra of Studied Compounds

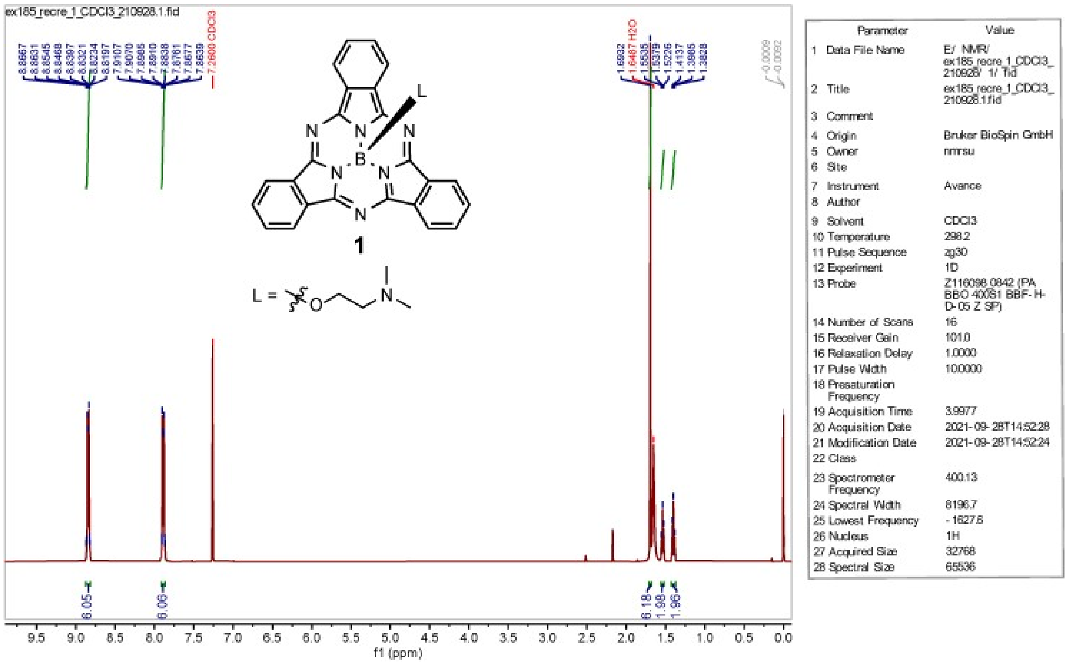

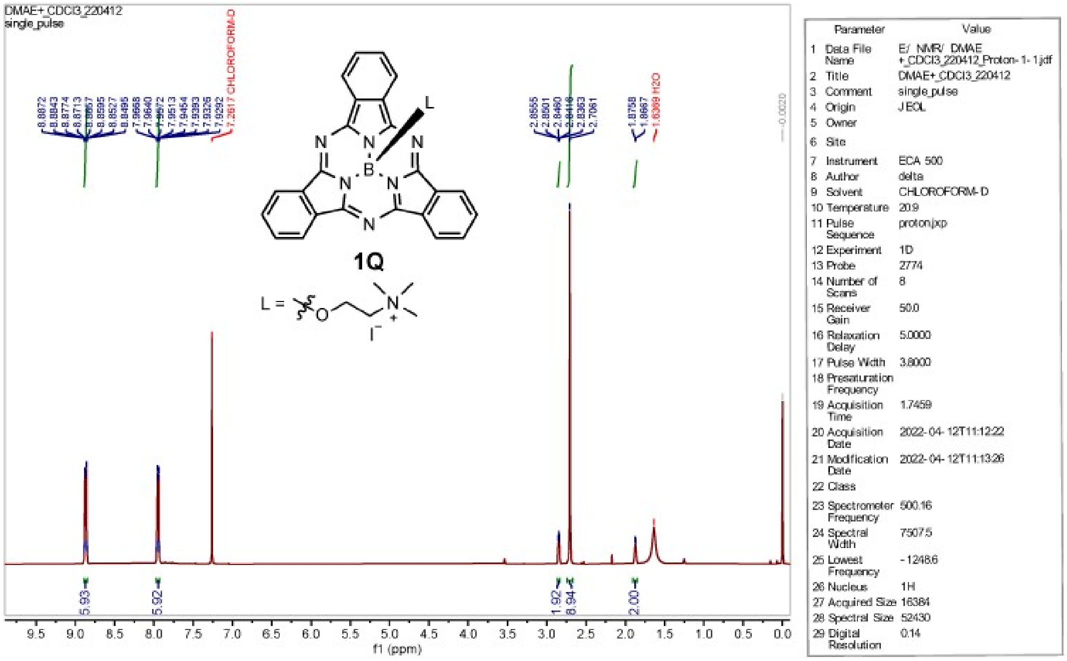

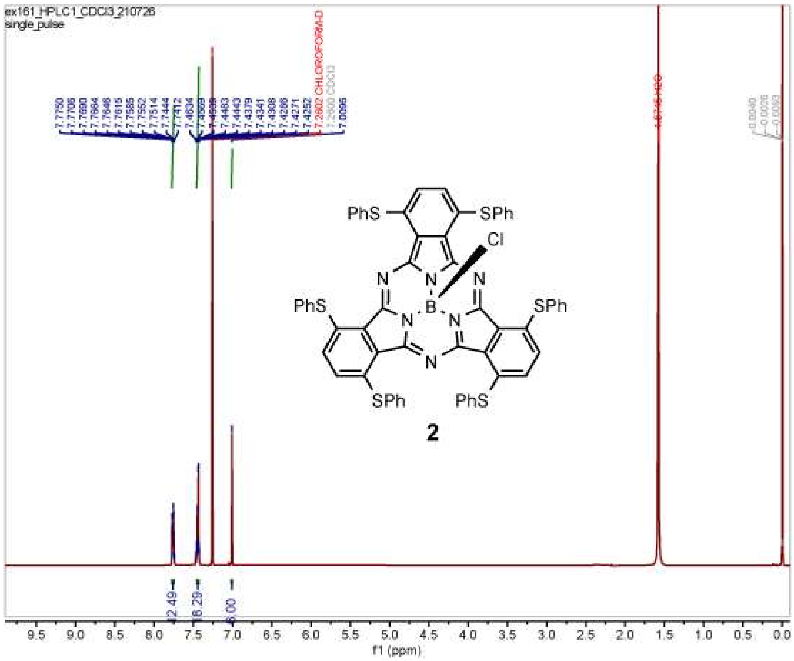

Parameter	Value
1 Data File Name	J:/ NMR/ ex161_HPLC1_CDCl3_210726_proton-1-1.jdf
2 Title	ex161_HPLC1_CDCl3_210726
3 Comment	single_pulse
4 Origin	JEOL
5 Owner	
6 Site	
7 Instrument	ECA 500
8 Author	delta
9 Solvent	CHLOROFORM-D
10 Temperature	21.2
11 Pulse Sequence	proton.jxp
12 Experiment	1D
13 Probe	2756
14 Number of Scans	8
15 Receiver Gain	54.0
16 Relaxation Delay	5.0000
17 Pulse Width	5.4500
18 Presaturation Frequency	
19 Acquisition Time	1.7459
20 Acquisition Date	2021-07-26T09:14:21
21 Modification Date	2021-07-26T09:15:23
22 Class	
23 Spectrometer Frequency	500.16
24 Spectral Width	7507.5
25 Lowest Frequency	-1253.0
26 Nucleus	1H
27 Acquired Size	16384
28 Spectral Size	52430

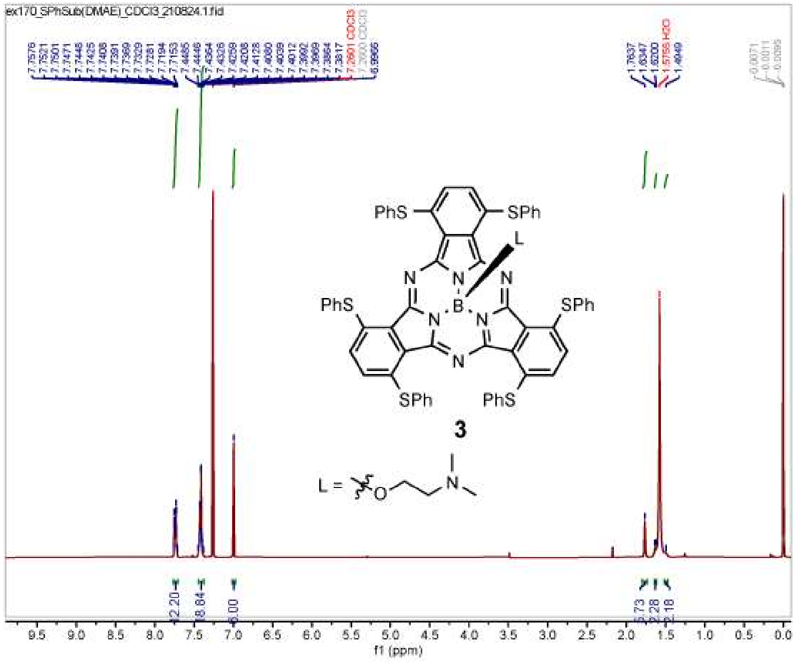

Parameter	Value
1 Data File Name	J:/ NMR/ ex170_SPhSub(DMAE)_CDCl3_210824/ 1/ fid
2 Title	ex170_SPhSub(DMAE)_CDCl3_210824.1.fid
3 Comment	
4 Origin	Bruker BioSpin GmbH
5 Owner	nmrsu
6 Site	
7 Instrument	Avance
8 Author	
9 Solvent	CDCl3
10 Temperature	299.8
11 Pulse Sequence	zg30
12 Experiment	1D
13 Probe	Z116098_0842 (PA BBO 400S1 BBF-H-D-05 Z SP)
14 Number of Scans	16
15 Receiver Gain	101.0
16 Relaxation Delay	1.0000
17 Pulse Width	10.0000
18 Presaturation Frequency	
19 Acquisition Time	3.9977
20 Acquisition Date	2021-08-24T16:58:45
21 Modification Date	2021-08-24T16:58:46
22 Class	
23 Spectrometer Frequency	400.13
24 Spectral Width	8196.7
25 Lowest Frequency	-1627.6
26 Nucleus	1H
27 Acquired Size	32768
28 Spectral Size	65536

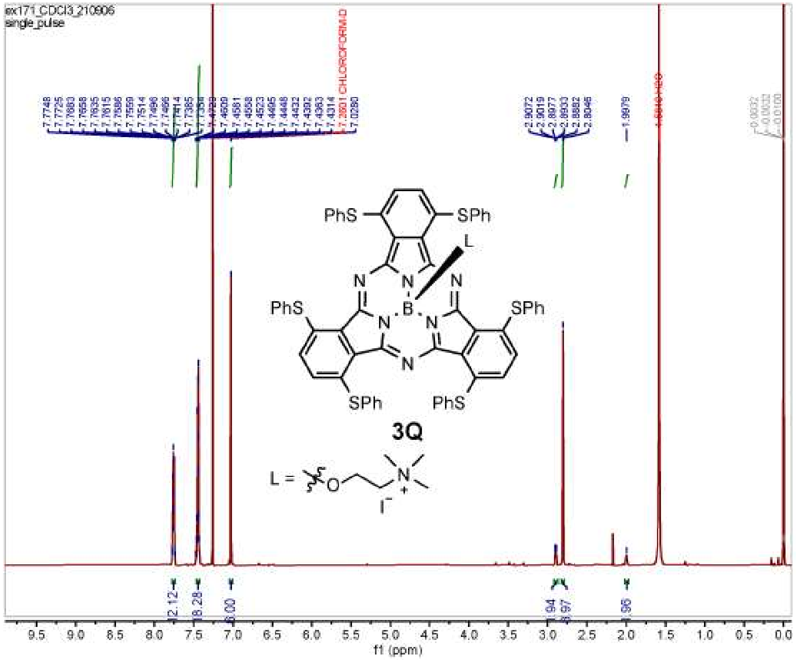

	Parameter	Value
1	Data File Name	J:/ NMR/ ex171_CDCl3_210906_proton-1-1.jdf
2	Title	ex171_CDCl3_210906
3	Comment	single_pulse
4	Origin	JEOL
5	Owner	
6	Site	
7	Instrument	ECA 500
8	Author	delta
9	Solvent	CHLOROFORM-D
10	Temperature	21.1
11	Pulse Sequence	proton.jxp
12	Experiment	1D
13	Probe	2756
14	Number of Scans	8
15	Receiver Gain	54.0
16	Relaxation Delay	5.0000
17	Pulse Width	5.4500
18	Presaturation Frequency	
19	Acquisition Time	1.7459
20	Acquisition Date	2021-09-06T12:54:03
21	Modification Date	2021-09-06T12:55:06
22	Class	
23	Spectrometer Frequency	500.16
24	Spectral Width	7507.5
25	Lowest Frequency	-1253.0
26	Nucleus	1H
27	Acquired Size	16384
28	Spectral Size	52430

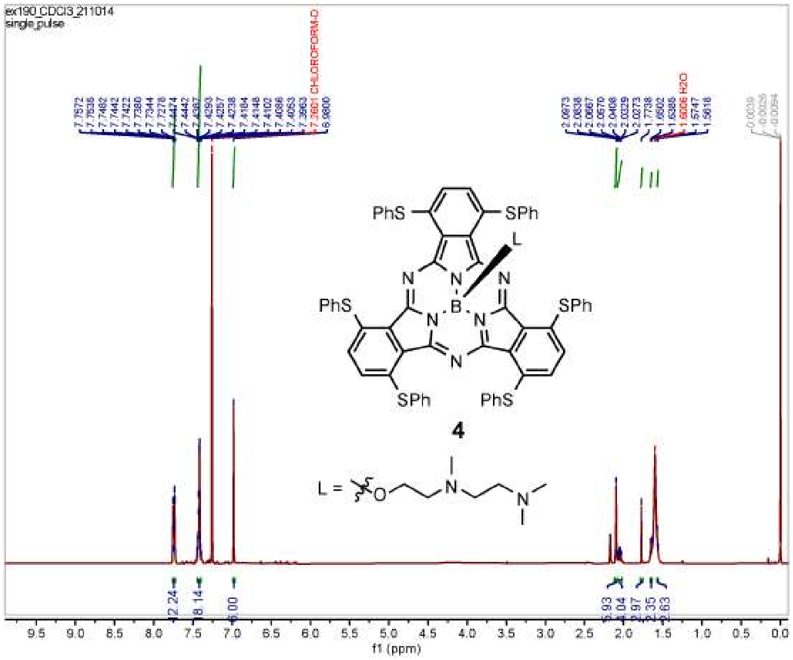

	Parameter	Value
1	Data File Name	E:/ NMR/ ex190_CDCl3_211014_proton-1-1.jdf
2	Title	ex190_CDCl3_211014
3	Comment	single_pulse
4	Origin	JEOL
5	Owner	
6	Site	
7	Instrument	ECA 500
8	Author	delta
9	Solvent	CHLOROFORM-D
10	Temperature	20.6
11	Pulse Sequence	proton.jxp
12	Experiment	1D
13	Probe	2756
14	Number of Scans	8
15	Receiver Gain	56.0
16	Relaxation Delay	5.0000
17	Pulse Width	5.4500
18	Presaturation Frequency	
19	Acquisition Time	1.7459
20	Acquisition Date	2021-10-14T09:03:14
21	Modification Date	2021-10-14T09:04:16
22	Class	
23	Spectrometer Frequency	500.16
24	Spectral Width	7507.5
25	Lowest Frequency	-1253.0
26	Nucleus	1H
27	Acquired Size	16384
28	Spectral Size	52430

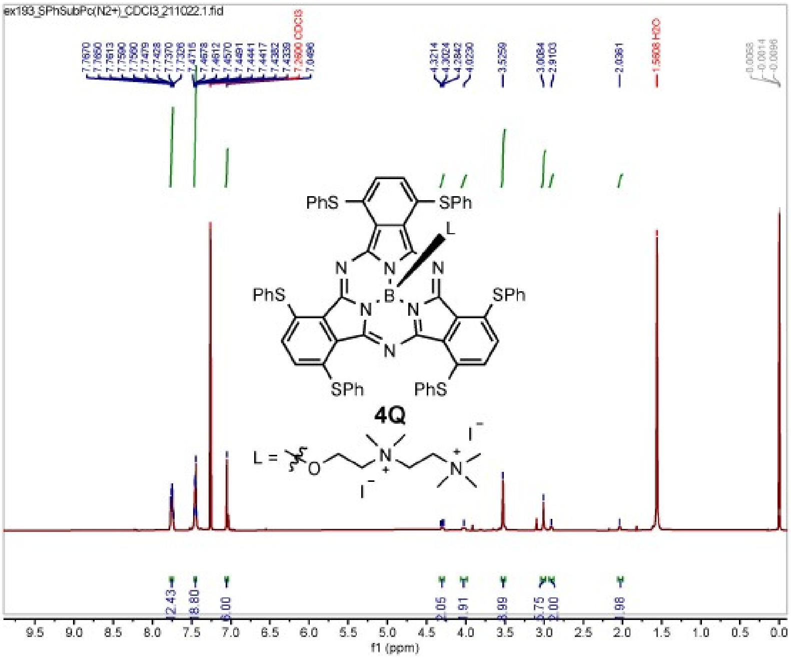

	Parameter	Value
1	Data File Name	E/ NMR/ ex193_SPhSubPc(N2+) _CDCl3_211022/ 1/ fid
2	Title	ex193_SPhSubPc(N2+) _CDCl3_211022.1.fid
3	Comment	
4	Origin	Bruker BioSpin GmbH
5	Owner	nmrsu
6	Site	
7	Instrument	Avance
8	Author	
9	Solvent	CDCl3
10	Temperature	298.2
11	Pulse Sequence	zg30
12	Experiment	1D
13	Probe	Z116098_0842 (PA BBO 400S1 BBF- H- D- 05 Z SP)
14	Number of Scans	16
15	Receiver Gain	101.0
16	Relaxation Delay	1.0000
17	Pulse Width	10.0000
18	Presaturation Frequency	
19	Acquisition Time	3.9977
20	Acquisition Date	2021- 10- 22T15:34:26
21	Modification Date	2021- 10- 22T15:34:22
22	Class	
23	Spectrometer Frequency	400.13
24	Spectral Width	8196.7
25	Lowest Frequency	- 1627.6
26	Nucleus	1H
27	Acquired Size	32768
28	Spectral Size	65536

Full Computational Details

Computational Details

Geometry optimization for all molecules was performed at the DFT level, by means of the hybrid Becke3LYP [36–39] (B3LYP) functional as implemented in Gaussian 16 [40]. The 6-31G(d) basis set was used for the all atoms. After the geometry optimization, the time-dependent (TD) DFT calculations [41,42] were performed to evaluate the stick absorption spectrum employing the same level or ωB97XD [43] and basis set. All calculations used a relatively simple self-consistent reaction field (SCRF) method based on the polarizable continuum model (PCM) [44] that mimicked the solvation effect of dichloromethane ($\varepsilon = 8.93$). All stationary points were optimized without any symmetry assumptions, and characterized by normal coordinate analysis at the same level of theory (the number of imaginary frequencies, NIMAG was 0).

Table S1. Calculated excited wavelengths (λ) and oscillator strengths (f) for components of selected transition energies. Calculations were performed at the ωB97XD/6-31G*//B3LYP/6-31G* level of theory using a polarizable continuum model (PCM), which mimicked the solvation effect of dichloromethane.

	λ / nm	f	Composition
**1**	504	0.46	HOMO-4→LUMO+1 (2.5%), HOMO→LUMO (94.9%)
	503	0.46	HOMO-4→LUMO (2.4%), HOMO→LUMO+1 (94.8%)
**1Q**	507	0.45	HOMO→LUMO (95.2%)
	504	0.45	HOMO→LUMO+1 (95.1%)
**3’**	553	0.57	HOMO→LUMO (93.1%)
	552	0.57	HOMO→LUMO+1 (93.0%)
**3Q’**	561	0.57	HOMO→LUMO (93.3%)
	558	0.57	HOMO→LUMO+1 (93.3%)

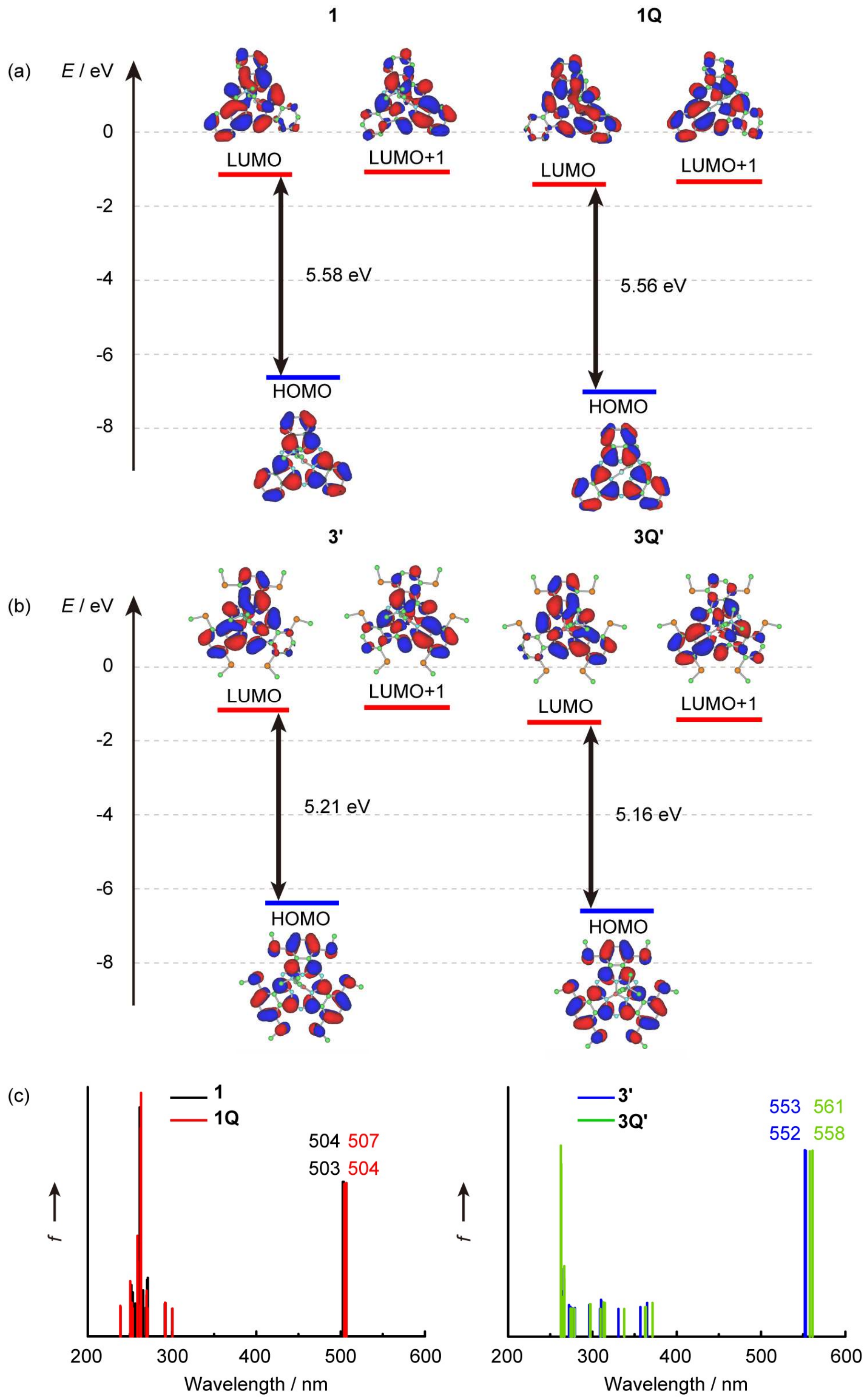

Figure S1. Partial molecular energy diagram and orbitals of (a) peripherally unsubstituted SubPcs (**1** and **1Q**), (b) peripherally substituted (MeS)$_6$SubPcs (**3'** and **3Q'**), and (c) their calculated absorption spectra. Calculations were performed at the ωB97XD/6-31G*//B3LYP/6-31G* level of theory, using a polarizable

continuum model (PCM), which mimicked the solvation effect of dichloromethane.

Cartesian Coordinates and Total Electron Energies

1

SCF Done: E(RB3LYP) = -1563.69370529 A.U.

Center Number	Atomic Number	Atomic Type	Coordinates (Angstroms) X	Y	Z
1	5	0	-0.169416	0.024748	0.627028
2	7	0	-0.190207	1.252469	-0.257152
3	6	0	-0.953279	1.352177	-1.389242
4	6	0	-0.686028	2.680105	-1.929668
5	6	0	-1.195068	3.364136	-3.036803
6	6	0	-0.665332	4.617232	-3.338535
7	1	0	-1.056741	5.174075	-4.184858
8	6	0	0.374463	5.174258	-2.568643
9	1	0	0.767331	6.151346	-2.834231
10	6	0	0.910328	4.491998	-1.478096
11	6	0	0.370650	3.246141	-1.147088
12	6	0	0.733712	2.256322	-0.139392
13	7	0	1.870045	2.112916	0.562270
14	7	0	1.288165	-0.160692	0.939115
15	6	0	2.167460	0.884053	1.016554
16	6	0	3.456491	0.287114	1.347123
17	6	0	4.714878	0.843453	1.591715
18	6	0	5.787399	-0.019913	1.806515
19	1	0	6.771173	0.390373	2.014876
20	6	0	5.621011	-1.417650	1.752895
21	1	0	6.479292	-2.061653	1.920752
22	6	0	4.377907	-1.986637	1.483112
23	6	0	3.287332	-1.133851	1.292707
24	6	0	1.897343	-1.385300	0.929773
25	7	0	1.324196	-2.472340	0.387026
26	7	0	-0.469811	-1.093814	-0.346628
27	6	0	0.192144	-2.292244	-0.313149
28	6	0	-0.383783	-3.090539	-1.389116
29	6	0	-0.148550	-4.399846	-1.817555
30	6	0	-0.820081	-4.853722	-2.951041
31	1	0	-0.665136	-5.872957	-3.292672
32	6	0	-1.693679	-4.012296	-3.667395
33	1	0	-2.197503	-4.396843	-4.549361
34	6	0	-1.917255	-2.696166	-3.267895
35	6	0	-1.271418	-2.235343	-2.117407
36	6	0	-1.224779	-0.926333	-1.476258
37	7	0	-1.543735	0.287207	-1.956722
38	8	0	-0.944835	0.071481	1.820528
39	6	0	-2.352880	0.241109	1.750316
40	1	0	-2.802604	-0.558060	1.143521
41	1	0	-2.614117	1.201158	1.281143
42	6	0	-2.896438	0.219305	3.180119
43	1	0	-2.619981	-0.742096	3.656349
44	1	0	-2.378362	1.008641	3.736473
45	7	0	-4.334436	0.478148	3.250294
46	6	0	-4.734678	0.867948	4.598909
47	1	0	-5.803378	1.109330	4.609562
48	1	0	-4.558904	0.074751	5.351941
49	1	0	-4.179570	1.759525	4.909390
50	6	0	-5.129520	-0.658604	2.792748
51	1	0	-4.972853	-1.566699	3.408000
52	1	0	-6.193214	-0.400605	2.834760
53	1	0	-4.888025	-0.907310	1.755420
54	1	0	-1.985426	2.929673	-3.640997

```
    55         1          0       1.722400    4.916221   -0.895816
    56         1          0       4.848811    1.920413    1.618046
    57         1          0       4.255162   -3.063768    1.426610
    58         1          0       0.536269   -5.045172   -1.276199
    59         1          0      -2.578484   -2.044622   -3.830710
---------------------------------------------------------------------
```

TD–DFT output
HOMO**: 126**, LUMO**: 127**

```
Excited State   1:      Singlet-A      2.4498 eV  506.10 nm  f=0.4147  <S**2>=0.000
     122 ->128         0.11055
     126 ->127         0.69569
 This state for optimization and/or second-order correction.
 Total Energy, E(TD-HF/TD-DFT) =  -1563.60367664
 Copying the excited state density for this state as the 1-particle RhoCI density.

 Excited State   2:      Singlet-A      2.4553 eV  504.97 nm  f=0.4143  <S**2>=0.000
     122 ->127        -0.11127
     126 ->128         0.69524

 Excited State   3:      Singlet-A      2.8262 eV  438.70 nm  f=0.0005  <S**2>=0.000
     125 ->127         0.70591

 Excited State   4:      Singlet-A      2.8477 eV  435.39 nm  f=0.0006  <S**2>=0.000
     125 ->128         0.70583

 Excited State   5:      Singlet-A      3.6344 eV  341.14 nm  f=0.0000  <S**2>=0.000
     123 ->128         0.36552
     124 ->127         0.57376
     126 ->131         0.12842

 Excited State   6:      Singlet-A      3.6960 eV  335.45 nm  f=0.0009  <S**2>=0.000
     122 ->127         0.12013
     123 ->127         0.44567
     124 ->128         0.47302
     126 ->129         0.15753
     126 ->130        -0.13851

 Excited State   7:      Singlet-A      3.7012 eV  334.99 nm  f=0.0015  <S**2>=0.000
     123 ->128         0.55165
     124 ->127        -0.34802
     126 ->129         0.13861
     126 ->130         0.15163

Excited State   8:      Singlet-A      3.8671 eV  320.62 nm  f=0.0187  <S**2>=0.000
     120 ->127        -0.16141
     121 ->128         0.20167
     123 ->127         0.45171
     124 ->128        -0.44795

 Excited State   9:      Singlet-A      3.8753 eV  319.93 nm  f=0.0212  <S**2>=0.000
     123 ->127        -0.14692
     126 ->129         0.64948

 Excited State  10:      Singlet-A      3.8776 eV  319.75 nm  f=0.0211  <S**2>=0.000
     123 ->128        -0.10046
     124 ->127         0.11043
     126 ->130         0.65340

 Excited State  11:      Singlet-A      3.9138 eV  316.79 nm  f=0.0000  <S**2>=0.000
     121 ->127         0.17249
     123 ->128        -0.11860
     126 ->131         0.65717

 Excited State  12:      Singlet-A      3.9838 eV  311.22 nm  f=0.0274  <S**2>=0.000
     119 ->127         0.56088
```

121 ->127 -0.34474
122 ->128 -0.12141
126 ->131 0.17365

Excited State 13: Singlet-A 4.0405 eV 306.85 nm f=0.3596 <S**2>=0.000
118 ->127 -0.16506
119 ->128 -0.24441
120 ->127 0.12838
122 ->127 0.59328
123 ->127 -0.13580

Excited State 14: Singlet-A 4.0464 eV 306.41 nm f=0.0113 <S**2>=0.000
115 ->128 0.10290
118 ->127 -0.11435
119 ->128 0.49831
120 ->127 -0.25162
121 ->128 -0.24123
122 ->127 0.26964

Excited State 15: Singlet-A 4.0514 eV 306.03 nm f=0.2756 <S**2>=0.000
118 ->128 -0.19470
121 ->127 -0.11052
122 ->128 0.64061

Excited State 16: Singlet-A 4.0799 eV 303.89 nm f=0.0005 <S**2>=0.000
116 ->128 0.12118
119 ->127 0.27175
120 ->128 0.50576
121 ->127 0.36468

Excited State 17: Singlet-A 4.0963 eV 302.68 nm f=0.0148 <S**2>=0.000
115 ->128 0.17354
116 ->127 0.18890
120 ->127 0.41591
121 ->128 -0.41060
123 ->127 0.17205
124 ->128 -0.15786

Excited State 18: Singlet-A 4.1242 eV 300.62 nm f=0.1869 <S**2>=0.000
116 ->128 0.12331
119 ->127 -0.29239
120 ->128 0.41903
121 ->127 -0.39062
126 ->130 0.10068

Excited State 19: Singlet-A 4.1307 eV 300.15 nm f=0.1471 <S**2>=0.000
116 ->127 0.11874
118 ->127 0.12941
119 ->128 0.41163
120 ->127 0.34773
121 ->128 0.36580

Excited State 20: Singlet-A 4.3135 eV 287.43 nm f=0.0591 <S**2>=0.000
115 ->127 0.39280
115 ->128 -0.10795
116 ->128 0.40651
118 ->128 0.36870

1Q

SCF Done: E(RB3LYP) = -1603.45188257 A.U.

Center Number	Atomic Number	Atomic Type	Coordinates (Angstroms) X	Y	Z
1	5	0	0.090501	-0.000953	-0.494225
2	7	0	0.076985	1.209193	0.403356
3	6	0	0.641002	1.225921	1.652602

4	6	0	0.396514	2.563771	2.176652
5	6	0	0.763360	3.185321	3.373673
6	6	0	0.294410	4.474424	3.617454
7	1	0	0.578883	4.982861	4.533931
8	6	0	-0.549703	5.129969	2.699514
9	1	0	-0.901330	6.132611	2.923948
10	6	0	-0.946032	4.512702	1.515036
11	6	0	-0.461425	3.230065	1.243653
12	6	0	-0.729570	2.291072	0.161647
13	7	0	-1.738161	2.255374	-0.724316
14	7	0	-1.292027	-0.054540	-1.063399
15	6	0	-2.056532	1.064386	-1.256738
16	6	0	-3.314967	0.585521	-1.816083
17	6	0	-4.463313	1.252847	-2.250528
18	6	0	-5.552104	0.488726	-2.665634
19	1	0	-6.449475	0.985568	-3.022292
20	6	0	-5.513913	-0.918791	-2.625264
21	1	0	-6.382474	-1.483122	-2.951538
22	6	0	-4.385996	-1.597795	-2.168728
23	6	0	-3.276139	-0.845489	-1.774984
24	6	0	-1.994523	-1.222564	-1.190914
25	7	0	-1.613057	-2.361922	-0.591253
26	7	0	0.140571	-1.155680	0.472119
27	6	0	-0.605701	-2.291885	0.294196
28	6	0	-0.289229	-3.150762	1.428421
29	6	0	-0.703880	-4.440043	1.774315
30	6	0	-0.276681	-4.965398	2.991887
31	1	0	-0.573674	-5.971081	3.274272
32	6	0	0.529042	-4.213066	3.869240
33	1	0	0.839160	-4.651596	4.813101
34	6	0	0.927701	-2.916695	3.550645
35	6	0	0.529637	-2.386097	2.320264
36	6	0	0.702622	-1.069654	1.719397
37	7	0	1.035730	0.105214	2.279212
38	8	0	1.069029	-0.006071	-1.551505
39	6	0	2.440517	0.033789	-1.234098
40	1	0	2.720912	-0.805959	-0.583677
41	1	0	2.698829	0.966861	-0.714660
42	6	0	3.150511	-0.062626	-2.588603
43	1	0	2.885252	-1.007057	-3.068042
44	1	0	2.819120	0.757558	-3.229010
45	6	0	5.171875	-0.153461	-3.971852
46	1	0	6.261363	-0.116094	-3.962199
47	1	0	4.833284	-1.112518	-4.364240
48	1	0	4.773107	0.662373	-4.574626
49	6	0	5.239209	-1.123886	-1.721716
50	1	0	4.851773	-2.070343	-2.099722
51	1	0	6.325840	-1.100736	-1.806918
52	1	0	4.948154	-0.985943	-0.681801
53	6	0	5.148179	1.322051	-2.013665
54	1	0	4.696906	2.122140	-2.600778
55	1	0	4.858715	1.413045	-0.968210
56	1	0	6.234153	1.359540	-2.099031
57	7	0	4.671373	-0.002605	-2.555223
58	1	0	-1.338772	-5.015350	1.107840
59	1	0	1.533976	-2.332783	4.236079
60	1	0	1.399097	2.675657	4.090915
61	1	0	-1.610405	5.012903	0.817411
62	1	0	-4.501192	2.337542	-2.266868
63	1	0	-4.365067	-2.682101	-2.122932

TD–DFT output
HOMO**: 130**, LUMO**: 131**

Excited State 1: Singlet-A 2.4355 eV 509.06 nm f=0.4096 <S**2>=0.000
127 ->132 0.11111

```
      130 ->131         0.69588
 This state for optimization and/or second-order correction.
 Total Energy, E(TD-HF/TD-DFT) =  -1603.36237802
 Copying the excited state density for this state as the 1-particle RhoCI density.

Excited State   2:      Singlet-A      2.4450 eV  507.10 nm  f=0.4060  <S**2>=0.000
      127 ->131        -0.11152
      130 ->132         0.69541

Excited State   3:      Singlet-A      3.6280 eV  341.74 nm  f=0.0000  <S**2>=0.000
      128 ->131         0.51732
      129 ->132         0.45414
      130 ->135        -0.11835

Excited State   4:      Singlet-A      3.6873 eV  336.25 nm  f=0.0018  <S**2>=0.000
      128 ->131        -0.41848
      128 ->132         0.11013
      129 ->131         0.11373
      129 ->132         0.49185
      130 ->134        -0.17704

Excited State   5:      Singlet-A      3.6909 eV  335.92 nm  f=0.0008  <S**2>=0.000
      127 ->131        -0.15206
      128 ->131         0.11074
      128 ->132         0.42659
      129 ->131         0.46671
      129 ->132        -0.11146
      130 ->133         0.19124

Excited State   6:      Singlet-A      3.8561 eV  321.52 nm  f=0.0138  <S**2>=0.000
      125 ->131        -0.18734
      126 ->132        -0.21901
      128 ->132         0.45175
      129 ->131        -0.44314

 Excited State   7:      Singlet-A      3.8904 eV  318.69 nm  f=0.0121  <S**2>=0.000
      125 ->131         0.18217
      126 ->132        -0.12471
      128 ->132        -0.16564
      130 ->133         0.64030

 Excited State   8:      Singlet-A      3.8938 eV  318.42 nm  f=0.0135  <S**2>=0.000
      125 ->132        -0.15995
      126 ->131        -0.13759
      128 ->131        -0.13006
      130 ->134         0.64896

 Excited State   9:      Singlet-A      3.9370 eV  314.92 nm  f=0.0001  <S**2>=0.000
      126 ->131        -0.25023
      130 ->135         0.63936

 Excited State  10:      Singlet-A      4.0005 eV  309.92 nm  f=0.3890  <S**2>=0.000
      124 ->131         0.14918
      127 ->131         0.65802
      128 ->132         0.11823
      129 ->131         0.12035

 Excited State  11:      Singlet-A      4.0171 eV  308.64 nm  f=0.3514  <S**2>=0.000
      124 ->132         0.14069
      127 ->132         0.65731

Excited State  12:      Singlet-A      4.0261 eV  307.95 nm  f=0.0004  <S**2>=0.000
      123 ->131        -0.11048
      125 ->132        -0.31210
      126 ->131         0.53200
      127 ->132         0.13249
      130 ->135         0.25431
```

Excited State 13: Singlet-A 4.0705 eV 304.59 nm f=0.0737 <S**2>=0.000
120 ->132 0.14248
123 ->132 -0.16680
126 ->132 0.60172
129 ->131 -0.19161
130 ->133 0.12490

Excited State 14: Singlet-A 4.0767 eV 304.13 nm f=0.0839 <S**2>=0.000
121 ->131 -0.20063
125 ->131 0.60615
128 ->132 0.19222
130 ->133 -0.14467

Excited State 15: Singlet-A 4.0824 eV 303.70 nm f=0.1625 <S**2>=0.000
121 ->132 -0.14333
125 ->132 0.56785
126 ->131 0.28482
130 ->134 0.18012

Excited State 16: Singlet-A 4.2784 eV 289.79 nm f=0.0236 <S**2>=0.000
120 ->132 -0.28179
121 ->131 0.23801
122 ->132 0.20491
123 ->132 0.49988
126 ->132 0.19337

Excited State 17: Singlet-A 4.2866 eV 289.23 nm f=0.0136 <S**2>=0.000
120 ->131 -0.23469
121 ->132 0.20345
122 ->131 0.14377
123 ->131 0.56862
124 ->132 0.14327
126 ->131 0.15983

Excited State 18: Singlet-A 4.3130 eV 287.46 nm f=0.0096 <S**2>=0.000
120 ->132 0.10523
121 ->131 0.62275
122 ->132 -0.11514
123 ->132 -0.19744
124 ->131 -0.10078
125 ->131 0.16273

Excited State 19: Singlet-A 4.3611 eV 284.30 nm f=0.0044 <S**2>=0.000
121 ->132 0.61895
122 ->131 -0.11188
123 ->131 -0.22965
124 ->132 0.15422
125 ->132 0.12629

Excited State 20: Singlet-A 4.4628 eV 277.82 nm f=0.0927 <S**2>=0.000
120 ->131 0.24059
121 ->132 -0.12094
122 ->131 -0.39458
123 ->131 0.15749
124 ->132 0.45233

3'

SCF Done: E(RB3LYP) = -4188.68073062 A.U.

Center Number	Atomic Number	Atomic Type	Coordinates (Angstroms) X	Y	Z
1	5	0	0.012470	0.002721	1.197531
2	7	0	-0.335877	1.292471	0.492417
3	6	0	-1.549695	1.528416	-0.087866
4	6	0	-1.481018	2.884623	-0.616242

5	6	0	-2.442177	3.694265	-1.251646
6	6	0	-2.010675	4.953561	-1.685065
7	1	0	-2.704159	5.631911	-2.167360
8	6	0	-0.688847	5.388179	-1.520191
9	1	0	-0.438384	6.377294	-1.884414
10	6	0	0.289383	4.592264	-0.912120
11	6	0	-0.125556	3.328727	-0.448654
12	6	0	0.604521	2.232731	0.177139
13	7	0	1.922783	1.967503	0.212919
14	7	0	1.405641	-0.290477	0.725717
15	6	0	2.298787	0.689464	0.401335
16	6	0	3.529645	-0.003588	0.040212
17	6	0	4.808123	0.470908	-0.311583
18	6	0	5.760907	-0.494905	-0.657277
19	1	0	6.768204	-0.197008	-0.922509
20	6	0	5.464740	-1.864312	-0.677488
21	1	0	6.260100	-2.544326	-0.958067
22	6	0	4.196202	-2.358879	-0.351911
23	6	0	3.227168	-1.407906	0.021779
24	6	0	1.819658	-1.542505	0.374266
25	7	0	0.954396	-2.549811	0.159864
26	7	0	-0.831216	-1.016042	0.467363
27	6	0	-0.356283	-2.251275	0.125982
28	6	0	-1.466360	-2.938382	-0.521579
29	6	0	-1.598670	-4.249880	-1.017476
30	6	0	-2.812946	-4.563532	-1.639672
31	1	0	-2.984888	-5.559557	-2.029692
32	6	0	-3.842501	-3.624306	-1.786842
33	1	0	-4.749775	-3.949806	-2.281499
34	6	0	-3.725727	-2.308936	-1.321923
35	6	0	-2.521284	-1.975793	-0.672609
36	6	0	-2.032269	-0.721845	-0.113351
37	7	0	-2.438441	0.544644	-0.314743
38	16	0	3.764456	-4.083108	-0.374611
39	16	0	5.130582	2.218620	-0.280934
40	16	0	1.980176	5.092356	-0.689312
41	16	0	-4.102150	3.089153	-1.448770
42	16	0	-4.994040	-1.075601	-1.495250
43	16	0	-0.257156	-5.398952	-0.816673
44	6	0	-0.963213	-6.967239	-1.427714
45	1	0	-1.199192	-6.918790	-2.493623
46	1	0	-0.177659	-7.711332	-1.274624
47	1	0	-1.846802	-7.260934	-0.855412
48	6	0	-6.382599	-2.010980	-2.221679
49	1	0	-7.199961	-1.290085	-2.301564
50	1	0	-6.142633	-2.383494	-3.220584
51	1	0	-6.696655	-2.833528	-1.574232
52	6	0	-4.986021	4.529030	-2.139323
53	1	0	-4.611807	4.798564	-3.130064
54	1	0	-6.026129	4.206685	-2.231319
55	1	0	-4.939242	5.390101	-1.467894
56	6	0	1.979084	6.822826	-1.269018
57	1	0	2.999046	7.178438	-1.103735
58	1	0	1.749732	6.894092	-2.335101
59	1	0	1.289177	7.441097	-0.689222
60	6	0	6.921406	2.314364	-0.619305
61	1	0	7.166090	1.951283	-1.620563
62	1	0	7.165004	3.378058	-0.561184
63	1	0	7.502287	1.775390	0.133355
64	6	0	5.356233	-4.901353	-0.732205
65	1	0	5.733489	-4.640484	-1.724099
66	1	0	6.105290	-4.675694	0.030991
67	1	0	5.137131	-5.971686	-0.709120
68	8	0	-0.054925	0.001592	2.619673
69	6	0	-1.284618	0.267657	3.278159
70	1	0	-2.057582	-0.438023	2.941215
71	1	0	-1.642276	1.283628	3.054906

72	6	0	-1.041464	0.141394	4.783221
73	1	0	-0.675069	-0.880638	5.004109
74	1	0	-0.229311	0.831109	5.039452
75	7	0	-2.211911	0.495010	5.586070
76	6	0	-1.838389	0.761839	6.971629
77	1	0	-2.722545	1.078549	7.536014
78	1	0	-1.407112	-0.120433	7.484359
79	1	0	-1.100571	1.570349	7.009621
80	6	0	-3.257735	-0.523048	5.528321
81	1	0	-2.924134	-1.503375	5.922669
82	1	0	-4.119006	-0.195338	6.120475
83	1	0	-3.597981	-0.670441	4.499395

TD–DFT output
HOMO**: 198**, LUMO**: 199**

Excited State 1: Singlet-A 2.0201 eV 613.76 nm f=0.4299 <S**2>=0.000
198 ->199 0.70092
This state for optimization and/or second-order correction.
Total Energy, E(TD-HF/TD-DFT) = -4188.60649362
Copying the excited state density for this state as the 1-particle RhoCI density.

Excited State 2: Singlet-A 2.0268 eV 611.72 nm f=0.4324 <S**2>=0.000
198 ->200 0.70085

Excited State 3: Singlet-A 2.5625 eV 483.84 nm f=0.0176 <S**2>=0.000
196 ->199 0.10768
197 ->199 0.69411

Excited State 4: Singlet-A 2.5689 eV 482.63 nm f=0.0304 <S**2>=0.000
196 ->199 0.51049
197 ->200 -0.48362

Excited State 5: Singlet-A 2.5760 eV 481.31 nm f=0.0132 <S**2>=0.000
196 ->200 0.68904
197 ->200 -0.12412
Excited State 6: Singlet-A 2.6297 eV 471.47 nm f=0.0142 <S**2>=0.000
196 ->199 0.46420
196 ->200 0.14247
197 ->199 -0.11621
197 ->200 0.49291

Excited State 7: Singlet-A 2.7580 eV 449.55 nm f=0.0003 <S**2>=0.000
195 ->199 0.70636

Excited State 8: Singlet-A 2.7797 eV 446.03 nm f=0.0003 <S**2>=0.000
195 ->200 0.70629

Excited State 9: Singlet-A 3.1742 eV 390.60 nm f=0.0492 <S**2>=0.000
188 ->200 0.11837
194 ->199 0.68585

Excited State 10: Singlet-A 3.1799 eV 389.90 nm f=0.0467 <S**2>=0.000
188 ->199 -0.11908
194 ->200 0.68561

Excited State 11: Singlet-A 3.4652 eV 357.80 nm f=0.0004 <S**2>=0.000
189 ->200 0.11829
190 ->199 -0.14044

192 ->199 0.13261
192 ->200 0.39346
193 ->199 0.51197
193 ->200 -0.12620

Excited State 12: Singlet-A 3.5217 eV 352.06 nm f=0.0242 <S**2>=0.000
189 ->199 0.10563
190 ->200 -0.10293
191 ->199 -0.17628
192 ->199 0.41737
192 ->200 0.16114
193 ->199 -0.13072
193 ->200 0.40400
198 ->201 -0.14912
198 ->202 0.17587

Excited State 13: Singlet-A 3.5254 eV 351.69 nm f=0.0204 <S**2>=0.000
189 ->200 0.12180
191 ->200 0.16142
192 ->199 -0.12640
192 ->200 0.46521
193 ->199 -0.34970
193 ->200 -0.16123
198 ->201 0.18371
198 ->202 0.14906

Excited State 14: Singlet-A 3.6378 eV 340.82 nm f=0.0077 <S**2>=0.000
192 ->199 0.19043
193 ->200 0.12117
198 ->201 0.63683
198 ->202 -0.12449

Excited State 15: Singlet-A 3.6397 eV 340.64 nm f=0.0067 <S**2>=0.000
192 ->200 -0.15129
193 ->199 0.16423
198 ->201 0.12375
198 ->202 0.63822

Excited State 16: Singlet-A 3.6625 eV 338.53 nm f=0.0077 <S**2>=0.000
192 ->199 -0.45983
192 ->200 0.14071
193 ->199 0.13208
193 ->200 0.49094

Excited State 17: Singlet-A 3.7195 eV 333.34 nm f=0.0000 <S**2>=0.000
189 ->200 -0.11221
190 ->199 0.10412
198 ->203 0.67202

Excited State 18: Singlet-A 3.7595 eV 329.79 nm f=0.0008 <S**2>=0.000
191 ->199 0.65609
192 ->199 0.14144
193 ->200 0.13230

Excited State 19: Singlet-A 3.7663 eV 329.19 nm f=0.0004 <S**2>=0.000
188 ->200 0.10483
190 ->199 -0.10888
191 ->200 0.65661

192 ->200	-0.12869
193 ->199	0.11449

Excited State 20: Singlet-A 3.8340 eV 323.38 nm f=0.0000 <S**2>=0.000

187 ->199	0.15808
189 ->200	-0.24767
190 ->199	0.59120
193 ->199	0.14080
198 ->203	-0.15387

3Q'

SCF Done: E(RB3LYP) = -4228.44003253 A.U.

Center Number	Atomic Number	Atomic Type	Coordinates (Angstroms) X	Y	Z
1	5	0	-0.020118	0.000442	1.042665
2	7	0	0.556614	-1.180260	0.311725
3	6	0	1.766097	-1.152701	-0.326547
4	6	0	1.950569	-2.487372	-0.880359
5	6	0	3.025152	-3.075784	-1.575539
6	6	0	2.838995	-4.391298	-2.016967
7	1	0	3.631515	-4.906212	-2.546434
8	6	0	1.644411	-5.090659	-1.801602
9	1	0	1.584312	-6.105426	-2.176064
10	6	0	0.554740	-4.521067	-1.132002
11	6	0	0.723730	-3.203694	-0.662623
12	6	0	-0.186006	-2.290507	0.015988
13	7	0	-1.527417	-2.301663	0.114663
14	7	0	-1.461599	0.007690	0.652524
15	6	0	-2.149344	-1.131578	0.345290
16	6	0	-3.511807	-0.701535	0.058353
17	6	0	-4.679603	-1.426038	-0.249344
18	6	0	-5.827615	-0.673013	-0.525319
19	1	0	-6.762905	-1.169037	-0.755034
20	6	0	-5.822937	0.727472	-0.519358
21	1	0	-6.755017	1.231606	-0.744398
22	6	0	-4.669887	1.470651	-0.237604
23	6	0	-3.506618	0.736050	0.063242
24	6	0	-2.140637	1.154078	0.352173
25	7	0	-1.509477	2.320462	0.127830
26	7	0	0.565562	1.181752	0.319883
27	6	0	-0.168065	2.299455	0.030372
28	6	0	0.750832	3.211408	-0.637756
29	6	0	0.592483	4.532617	-1.099941
30	6	0	1.690758	5.101366	-1.756218
31	1	0	1.638464	6.118582	-2.125145
32	6	0	2.884959	4.398811	-1.962929
33	1	0	3.685773	4.914301	-2.479214
34	6	0	3.060827	3.079604	-1.528363
35	6	0	1.975077	2.489795	-0.852369
36	6	0	1.777243	1.150758	-0.313727
37	7	0	2.423739	-0.002837	-0.562284
38	16	0	-4.611245	3.246406	-0.226930
39	16	0	-4.631697	-3.202052	-0.255178
40	16	0	-0.981402	-5.363735	-0.838097
41	16	0	4.516056	-2.143664	-1.831600
42	16	0	4.554518	2.146533	-1.764467
43	16	0	-0.942343	5.380219	-0.814927
44	6	0	-0.601005	7.068476	-1.418533
45	1	0	-0.408570	7.083855	-2.494079
46	1	0	-1.515058	7.632124	-1.216254
47	1	0	0.228163	7.530077	-0.876484
48	6	0	5.692142	3.366393	-2.505339
49	1	0	6.640777	2.836440	-2.620792

50	1	0	5.349710	3.693870	-3.489953
51	1	0	5.843191	4.227057	-1.848977
52	6	0	5.635736	-3.357968	-2.608098
53	1	0	5.270814	-3.676584	-3.587543
54	1	0	6.581924	-2.827570	-2.740320
55	1	0	5.801074	-4.224399	-1.962852
56	6	0	-0.650486	-7.051631	-1.448568
57	1	0	-1.562743	-7.614237	-1.235617
58	1	0	-0.471621	-7.065866	-2.526468
59	1	0	0.184773	-7.515124	-0.917582
60	6	0	-6.376757	-3.662835	-0.525976
61	1	0	-6.735525	-3.336851	-1.505296
62	1	0	-6.389991	-4.754964	-0.491795
63	1	0	-7.023125	-3.274979	0.265280
64	6	0	-6.353323	3.720476	-0.493739
65	1	0	-6.714021	3.405331	-1.475901
66	1	0	-7.002133	3.329745	0.294106
67	1	0	-6.359694	4.812320	-0.449996
68	8	0	0.111596	-0.003859	2.476857
69	6	0	1.391536	-0.039123	3.063449
70	1	0	2.001247	0.814533	2.736403
71	1	0	1.924910	-0.960629	2.792722
72	6	0	1.120864	0.024022	4.570430
73	1	0	0.621826	0.965425	4.809355
74	1	0	0.459825	-0.798820	4.850173
75	6	0	1.855034	0.036705	6.906649
76	1	0	2.716730	-0.017763	7.572069
77	1	0	1.339985	0.988749	7.034814
78	1	0	1.173139	-0.789942	7.105761
79	6	0	3.304588	1.058914	5.211794
80	1	0	2.776743	2.007472	5.313390
81	1	0	4.113590	0.999989	5.940248
82	1	0	3.707967	0.956746	4.205854
83	6	0	3.046238	-1.388835	5.308610
84	1	0	2.329404	-2.193811	5.472772
85	1	0	3.461689	-1.451123	4.304272
86	1	0	3.849956	-1.446545	6.042983
87	7	0	2.338431	-0.067639	5.480203

TD–DFT output
HOMO**: 202**, LUMO**: 203**

Excited State 1: Singlet-A 1.9743 eV 627.99 nm f=0.4190 <S**2>=0.000
202 ->203 0.70144
This state for optimization and/or second-order correction.
Total Energy, E(TD-HF/TD-DFT) = -4228.36747882
Copying the excited state density for this state as the 1-particle RhoCI density.

Excited State 2: Singlet-A 1.9866 eV 624.12 nm f=0.4223 <S**2>=0.000
202 ->204 0.70135

Excited State 3: Singlet-A 2.4900 eV 497.93 nm f=0.0202 <S**2>=0.000
201 ->203 0.70534

Excited State 4: Singlet-A 2.5070 eV 494.55 nm f=0.0317 <S**2>=0.000
200 ->203 0.49692
201 ->204 0.50022

Excited State 5: Singlet-A 2.5213 eV 491.75 nm f=0.0160 <S**2>=0.000
200 ->204 0.70501

Excited State 6: Singlet-A 2.5742 eV 481.64 nm f=0.0139 <S**2>=0.000
200 ->203 0.49783

201 ->204 -0.49429

Excited State 7: Singlet-A 3.1541 eV 393.09 nm f=0.0539 <S**2>=0.000
193 ->204 -0.11966
199 ->203 0.68624

Excited State 8: Singlet-A 3.1651 eV 391.73 nm f=0.0528 <S**2>=0.000
193 ->203 0.12147
199 ->204 0.68595

Excited State 9: Singlet-A 3.4357 eV 360.87 nm f=0.0000 <S**2>=0.000
194 ->203 0.13540
195 ->204 0.13306
197 ->203 0.47842
198 ->204 0.47064

Excited State 10: Singlet-A 3.4884 eV 355.42 nm f=0.0243 <S**2>=0.000
194 ->203 -0.10562
195 ->204 0.10825
196 ->204 0.16081
197 ->203 -0.44174
198 ->204 0.46333
202 ->206 0.17031

Excited State 11: Singlet-A 3.4944 eV 354.81 nm f=0.0257 <S**2>=0.000
194 ->204 0.10035
195 ->203 0.10388
196 ->203 -0.22116
197 ->204 0.39837
198 ->203 0.47155
202 ->205 -0.18778

Excited State 12: Singlet-A 3.6154 eV 342.94 nm f=0.0048 <S**2>=0.000
197 ->204 -0.40090
198 ->203 0.48557
202 ->205 0.30593

Excited State 13: Singlet-A 3.6259 eV 341.94 nm f=0.0055 <S**2>=0.000
196 ->203 -0.12710
197 ->204 0.34255
202 ->205 0.58681

Excited State 14: Singlet-A 3.6289 eV 341.66 nm f=0.0038 <S**2>=0.000
197 ->203 0.13605
198 ->204 -0.11534
202 ->206 0.66339

Excited State 15: Singlet-A 3.6960 eV 335.46 nm f=0.0008 <S**2>=0.000
196 ->203 0.64816
197 ->204 0.21218
198 ->203 0.13143

Excited State 16: Singlet-A 3.7121 eV 334.00 nm f=0.0002 <S**2>=0.000
195 ->204 0.17715
196 ->204 -0.45104
197 ->203 -0.16196
202 ->207 0.47035

Excited State 17:	Singlet-A	3.7192 eV	333.36 nm	f=0.0007	<S**2>=0.000
194 ->203	0.14057				
196 ->204	0.49534				
198 ->204	-0.15013				
202 ->207	0.43845				
Excited State 18:	Singlet-A	3.8169 eV	324.83 nm	f=0.0001	<S**2>=0.000
194 ->203	0.47695				
195 ->203	0.11262				
195 ->204	0.38444				
197 ->203	-0.11900				
202 ->207	-0.26756				
Excited State 19:	Singlet-A	3.8636 eV	320.90 nm	f=0.0014	<S**2>=0.000
194 ->203	0.25606				
194 ->204	0.32863				
195 ->203	0.36800				
195 ->204	-0.34421				
202 ->206	0.10225				
Excited State 20:	Singlet-A	3.8641 eV	320.86 nm	f=0.0011	<S**2>=0.000
193 ->203	-0.11422				
194 ->203	-0.33937				
194 ->204	0.26468				
195 ->203	0.33083				
195 ->204	0.36335				
